# Immunomodulatory effects of nematode-microbiota interactions in cancer therapy

**DOI:** 10.3389/fimmu.2026.1803276

**Published:** 2026-07-01

**Authors:** Valentyn Oksenych, Marjan Rashidan, Meisam Khazaei, Hamed Tahmasebi

**Affiliations:** 1 Faculty of Medicine, University of Bergen, Bergen, Norway; 2 Department of Microbiology, Danylo Halytsky Lviv National Medical University, Lviv, Ukraine; 3 Oslo Bioconsulting, Oslo, Norway; 4 School of Medicine, Shahroud University of Medical Sciences, Shahroud, Iran; 5 Department of Bacteriology, Pasteur Institute of Iran, Tehran, Iran

**Keywords:** cancer immunotherapy, excretory-secretory products, helminth therapy, microbiota, nematode, p43 protein, TM-DLP-1, *Trichuris muris*

## Abstract

Among parasites, roundworms release substances that quietly reshape how the body’s defences respond - a dance shaped by long evolution. One player, called p43 - also known as Tm-DLP-1 - (*Trichuris muris* Dorylaimia Lipid-carrying Protein-1), makes up nearly all of it. Scientists now see this protein not just as a worm tool but as something that can steer immune responses, possibly helping fight tumours. Here lies an exploration: how p43 alters immunity, teams up with gut microbes, and shows promise in early tests and trials against cancer. Beyond its role inside the parasite hauling lipids around, p43 works outside by grabbing tightly onto IL-13, a signal involved in fighting worms - and oddly enough - in feeding some cancers. Thanks to parts resembling the IL-13 receptor alpha-2 and sections like those found in thrombospondin, this protein binds IL-13 strongly while also attaching to sugar chains in the tissue scaffold, leaving pockets where immune behaviour shifts subtly. Despite strong results across several animal cancer models - slowed tumours, stronger CD8+ T cell attacks, weaker regulatory T cell activity - the path forward stays uncertain. Safety data in healthy humans come from trials using Trichuris suis ova, yet those with weakened immune systems face lingering risks. A molecule called p43/Tm-DLP-1 emerges here, not by chance, due to its ability to block IL-13 while shifting gut microbes. Still, progress demands more lab refinement, larger production methods, and human studies built with precision before any real impact can take hold.

## Graphical Abstract

**Figure:**
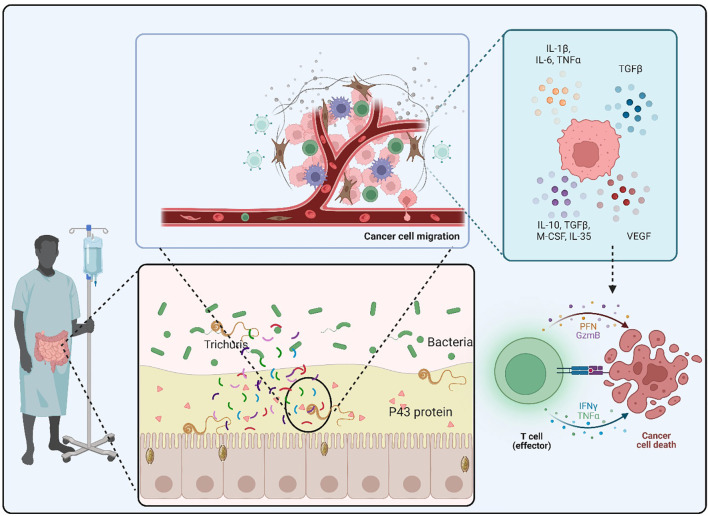

## 1 Introduction

### 1.1 Background and rationale

Cancer continues to be one of the leading causes of morbidity and mortality across the globe, accounting for 19.3 million new diagnoses of cancer each year and 10 million deaths due to cancer each year (1, 2). Standard therapies for treating cancer have improved significantly over time and include surgical, chemotherapeutic, radiation, targeted, and immune-based therapies; however, a sizable population continues to have an unmet medical need for these therapies. Immune checkpoint inhibitors (ICIs) and chimeric antigen receptor T-cell (CAR-T) therapies have dramatically changed the delivery of cancer care. However, many patients do not respond well to these therapies, and many develop resistance to them over time (2, 3). Additionally, new cancer drugs may result in significant financial toxicity for patients (4, 5). With the above in mind, there is a growing demand for additional affordable treatment options to meet public health needs.

The notion that helminths provide naturally occurring immunoglobulins and other regulatory factors to fight autoimmune diseases, as well as the potential for cancer prevention, has once again spurred research into helminth-derived immunomodulatory molecules (6, 7). The hygiene hypothesis, first proposed by Strachan (8), suggests that children in less hygienic conditions are exposed to more infectious agents, leading to a lower incidence of allergic diseases (which have increased substantially in developed countries) due to exposure to pathogens. This hypothesis has now grown to incorporate autoimmune diseases and cancers, potentially since epidemiological studies indicate that the incidence of helminthic infections inversely correlates with certain types of cancer (5, 9).

Soil-transmitted helminths such as *Ascaris lumbricoides*, *Trichuris trichiura*, *Ancylostoma duodenale* (hookworm), *Necator americanus* (hookworm), and *Strongyloides stercoralis* collectively infect approximately 1.5 billion people globally (10). *Trichuris* species have gained considerable interest (11) as they reside in epithelial cells, cause chronic infections, and possess robust immunomodulatory properties. Therefore, *T. muris* is the predominant model organism for studying host-parasite relationships. This has been demonstrated by collaborative research indicating that many traits are conserved among Trichuris species that infect multiple mammalian species (12).

Thus, nematode parasites release excretory-secretory products (ESPs) that contain numerous immunomodulatory factors that co-evolved with the mammalian immune system over millions of years (13, 14). These ESPs contain proteins, glycoproteins, lipids, nucleic acids, and extracellular vesicles, all of which are important for the parasite’s colonisation of the host (15). Of the ESPs produced by *T. muris*, protein p43 is the most prevalent as well as functionally important protein produced by adult parasites, accounting for at least 90% of the total secreted protein from adult *T. muris* (16).

New structural and functional analyses show that p43 is now named Tm-DLP-1 (*Trichuris muris* Dorylaimia Lipid-carrying Protein-1) and is a member of a new class of proteins called “dorylipophorins,” which are exclusively found in Nemata, Clade I nematodes (Dorylaimia) (17). p43 demonstrates the ability to perform two actions: it functions as the largest lipid transport protein (replacing both the major polyprotein allergens (NPAs) and fatty acid retinol-binding proteins (FARs) present in other nematode clades) within the pseudocoelomic fluid. It acts as a powerful IL-13 antagonist by binding IL-13 with high affinity and sequestering it (18, 19).

IL-13 is critical to cancer biology and has been shown to promote the survival, proliferation, and metastasis of cancer cells in many cancers, notably colorectal cancer, where it induces epithelial-mesenchymal transition and chemoresistance (20, 21). IL-13 also facilitates immune evasion by promoting regulatory T cell function while inhibiting cytotoxic CD8+ T cell function in the tumour microenvironment (22). Therefore, the ability of p43/Tm-DLP-1 to specifically neutralise IL-13 function makes it a viable target for cancer immunotherapy.

### 1.2 Scope and objectives

This review aims to provide a comprehensive analysis of the current evidence regarding nematode-microbiota interactions in cancer therapy, with particular emphasis on the immunomodulatory effects of the p43/Tm-DLP-1 protein from *Trichuris muris*. The specific objectives encompass: (i) detailed examination of the structural and functional properties of p43/Tm-DLP-1; (ii) elucidation of its molecular mechanisms of immunomodulation, particularly IL-13 inhibition; (iii) analysis of tripartite interactions between nematodes, gut microbiota, and host immunity; (iv) critical evaluation of preclinical animal trial data; (v) assessment of available human clinical evidence; and (vi) identification of challenges and future directions for therapeutic development.

## 2 Biology of *Trichuris muris* and the p43/Tm-DLP-1 protein

### 2.1 Taxonomic classification and life cycle

*T. muris* belongs to the Nematoda phylum, specifically the Enoplea class, Dorylaimia subclass, Trichocephalida order, and Trichuridae family (17, 23, 24). Unlike most commonly researched parasitic roundworms - such as Caenorhabditis elegans, Brugia malayi, and Ascaris suum - that sit in Clade III (Chromadoria), this organism is grouped in Clade I (Dorylaimia) (25). Because these two branches split nearly 500 million years ago, their biological traits have drifted apart noticeably (26). Genome layout, cellular functions, and their molecular-level regulation now differ quite markedly (26).

**Figure 1:**
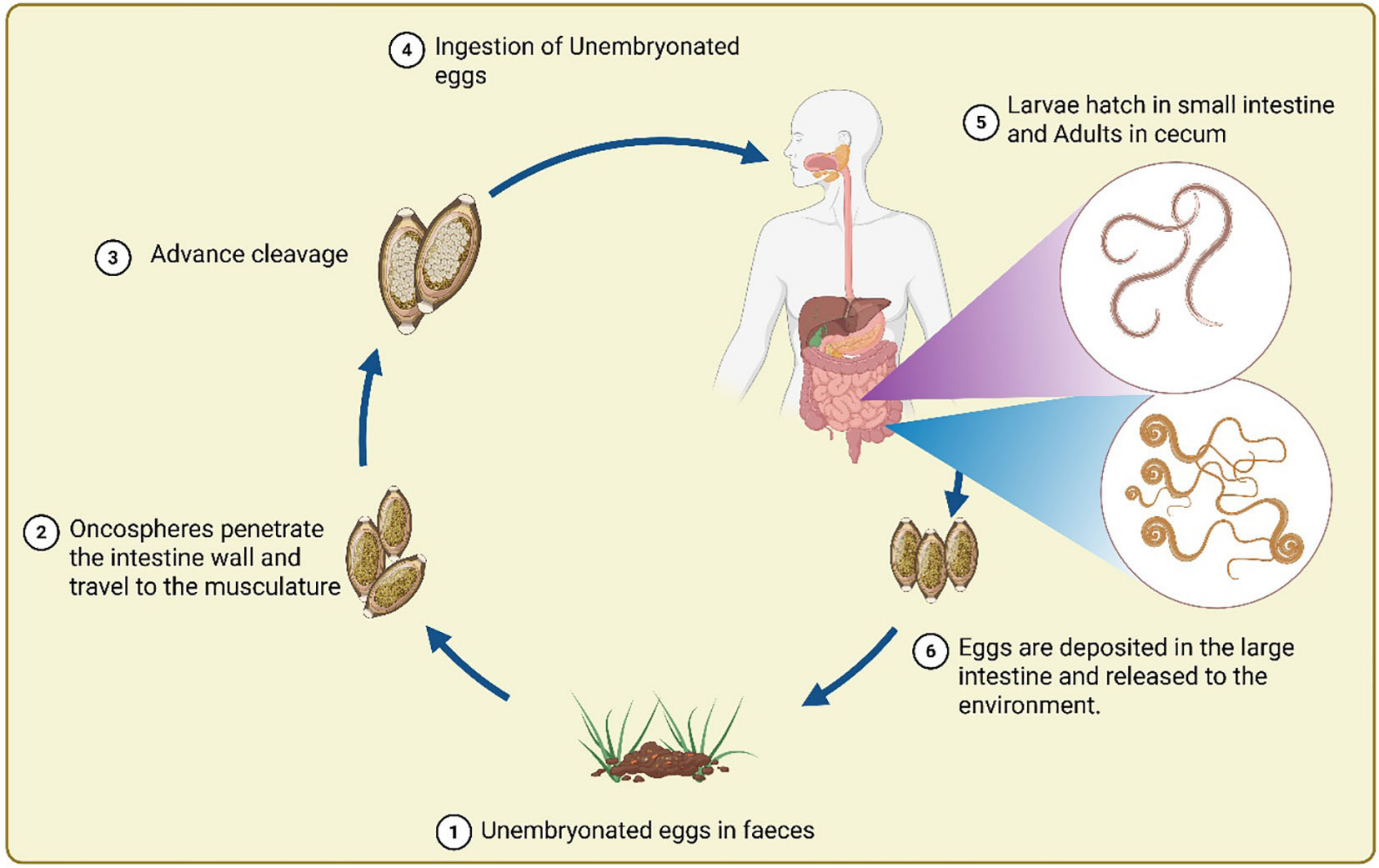
Life cycle of *T. muris*. Beginning at the top left, infection occurs via the faecal-oral route. Eggs hatch in the host caecum, releasing L1 larvae, which then burrow into the caecal epithelial crypts. Larvae undergo four moults to become adults at the time points specified on the diagram. Male and female worms mate, and eggs are released into the caecal lumen, where they are excreted in the faeces. Eggs must undergo embryonation before they become infective. L1/L2/L3/L4, First/second/third/fourth-stage larvae, respectively; p.i., post-infection. This figure was generated using BioRender software, version 04.

Larvae of *T. muris* follow a straightforward developmental path, needing just one host to finish their cycle. Inside durable eggs, infectious L1 stages enter the body when the eggs are consumed. Once they reach the cecum and upper colon, hatching begins in response to cues from gut microbes, especially Escherichia coli (27, 28). After emerging, these L1 forms swiftly invade the intestinal lining, embedding their anterior ends in cellular tunnels formed when several epithelial cells merge; meanwhile, their posterior ends remain exposed in the bowel lumen (29, 30). This specialised space within the epithelial layer positions *T. muris* close to both host cells and microbes in the gut lumen. Facing the intestinal contents, the front part of the worm - housing the bacillary band and stichosome - likely handles secretion and nutrient uptake needed for its survival (31). Found only in Trichuridae worms, the stichosome consists of several stichocyte cells joined by channels that directly link to the intestine; it may serve as the main source of excreted-secreted molecules (32) **Figure 1**.

In lab settings with heavy infection doses - about 200 to 400 eggs - the life cycle of *T. muris* unfolds in a consistent sequence. Within hours after being swallowed, L1 larvae emerge. Progressing slowly, they advance through the L2 and then L3 stages across roughly two to three weeks. By the 32nd to 35th day following infection, these parasites mature into adult or late-stage L4 forms (33–35). Around day 35, female worms start generating eggs. These undeveloped eggs exit via the host’s stool. From initial exposure to first egg release, this phase generally lasts between five and six weeks (35, 36). Under normal conditions, where small doses accumulate slowly over time, the body’s immune response changes in key ways. Instead of clearing worms quickly, repeated exposure leads to a gradual buildup, closely linked to an early Th1 response. Protection begins only later, once a more active Th2 response appears, as shown by Glover et al. (30). Because this shift takes time, mature parasites survive long enough to settle in before the defence fully kicks in. It may be precisely this window - when IL-13 activity lags - that p43 exploits to block protection.

### 2.2 Excretory-secretory products

Found within the space where parasites meet their hosts, secretions from parasitic roundworms form a shifting blend of substances. Because they help evade immune attacks, move through tissues, and acquire nutrients, these materials play key roles in the dialogue between host and invader (37, 38). Shaped by evolutionary adaptation to particular environments, what appears in these secretions varies across species, developmental phases, and external factors (39, 40).

A 2018 study led by Eichenberger examined adult excretory-secretory materials from *T. muris* using broad-scale protein detection methods, uncovering 148 distinct proteins - about one-third of those expected from its genome’s secretion profile (41, 42). When classified through gene function mapping and structural motif screening, most belonged to families resembling trypsin enzymes, thioredoxins, or those built around repeated four-amino-acid segments. Protein interactions, attachment to metal ions, and affinity for DNA or RNA emerged as prevalent roles among these molecules. Particularly interesting was the presence of SCP/TAPS-domain carriers - agents linked elsewhere in parasitic worms to immune system interference - though how they operate within Trichuris is still unclear (43, 44).

What sets *T. muris* ES apart from nearly every other examined nematode is not subtle - it is the overwhelming presence of one protein, p43. Instead of a mix, adult *T. muris* worms release p43 so abundantly that it makes up about 90–95 percent of their entire secreted protein output, far exceeding anything seen before in similar parasites (2, 13). Because such extreme concentration rarely happens by chance, researchers infer p43 plays an essential role - likely central to how the organism survives and remains within its host.

**Figure 2:**
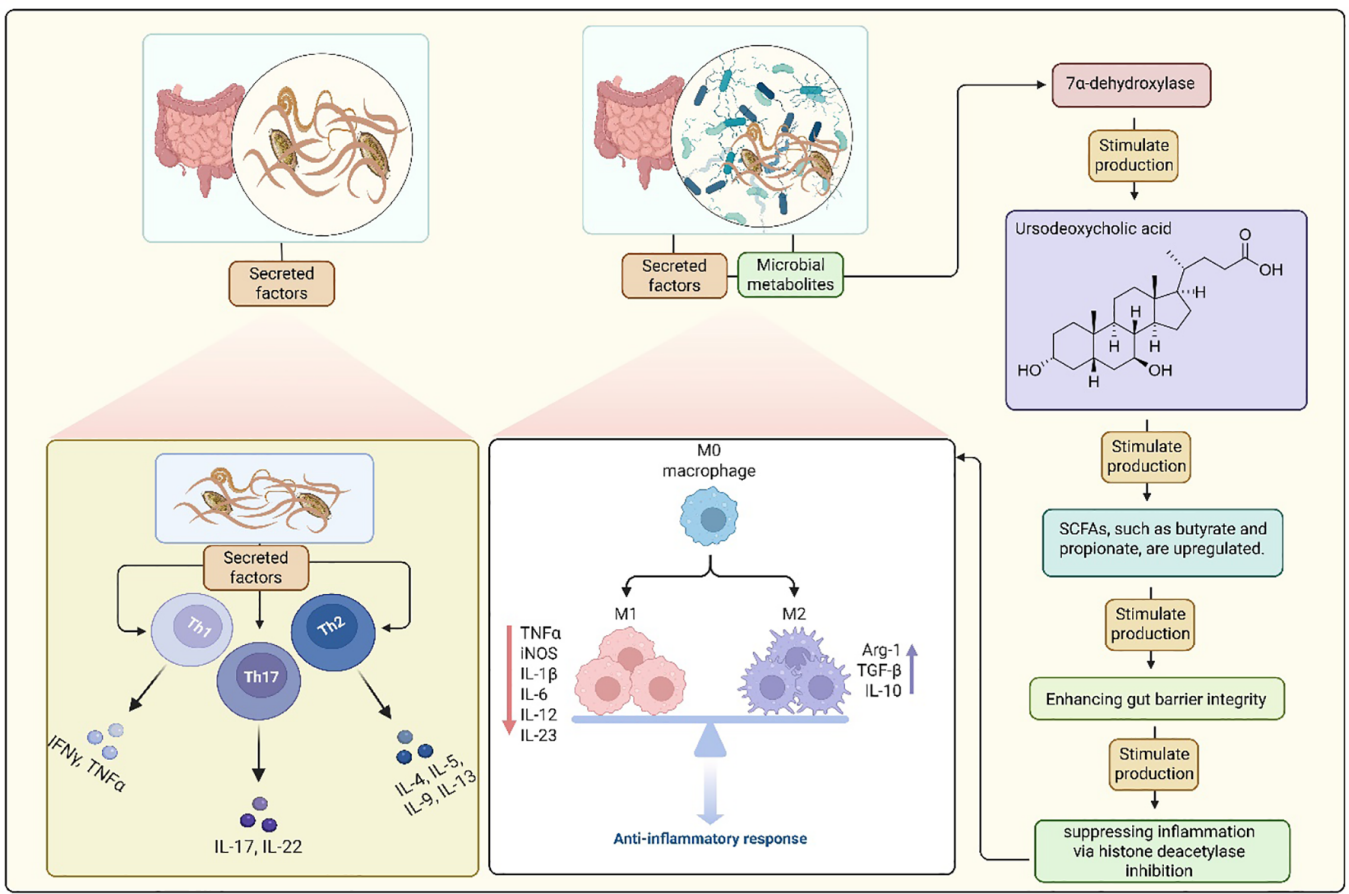
Worm eggs have a fascinating impact on the immune system by stimulating the production of Th1 and Th17 cells, whereas helminths generally induce a Th2 response. This difference in immune activation is partly due to the parasitic effectors released by these organisms, which play a crucial role in suppressing pro-inflammatory cytokines. In the complex ecosystem of the gut, members of the parasitome interact with commensal bacteria, secreting substances that could potentially enhance bacterial diversity. For instance, the parasite *Tritrichomonas musculis* activates inflammasomes in epithelial cells, leading to IL-18 production and prompting a Th1 and Th17 immune response. This activity fortifies the intestinal barrier against infections and limits bacterial invasion. *Heligmosomoides polygyrus* has been associated with increased levels of *S. Typhimurium*, though the mechanism by which it facilitates this colonisation remains unclear. Blood-associated parasites such as Plasmodium species and Schistosoma mansoni further complicate this dynamic by triggering a Th2 response, leading to IL-3 and IL-4 production that compromises the gut’s epithelial barrier. This disruption provides S. Typhimurium with an opportunity to exploit weakened defences, thereby enhancing its ability to invade epithelial cells. Understanding these interactions highlights the intricate balance between parasites and host immune responses in shaping gut health and disease susceptibility. This figure was generated using BioRender software, version 04.

Small molecules and metabolites are present in substantial amounts in *T. muris* ES, not just proteins. Identified through work by Wangchuk et al., fatty acids such as propionate, butyrate, and acetate - classified as SCFAs - are present alongside alanine and glutamine, among other amino acids. Fructose, galactose, and glucosamine turn up in sugar form, while adenine and uridine emerge as detectable nucleobases, all located in enriched ES portions (45, 46). Despite this metabolic profile, genetic analysis shows that *T. muris* lacks key genes required for the *de novo* synthesis of certain compounds, especially butyrate. This absence hints at an external origin - one possibility being microbial activity inside the worm’s digestive tract; another points toward uptake of substances originally made by the host organism (47–49) **Figure 2**.

Little spheres released by cells, such as those resembling exosomes, form a key part of what *T. muris* secretes into its surroundings. At first, work led by Tritten identified tiny amounts of particles in the fluid where adult worms grew, uncovering 14 distinct microRNAs and 73 likely worm-related proteins (50, 51). Later efforts by Eichenberger and then Shears added depth, listing 364 proteins and 56 microRNAs associated with these expelled bodies. When tested, these small packages reduced the strength of host systems’ detection of genetic material and dampened type I interferon responses in mini-gut tissues derived from mice. Surprisingly, even without extra boosting agents, using them for vaccination helped animals resist later infections (52, 53).

### 2.3 Discovery and characterisation of p43

Found in secretions from adult *T. muris* worms, the p43 protein stood out early due to how much of it appeared during lab tests. Though at first thought to resemble interferon-gamma - based on work led by Grencis and Entwistle back in 1997 - later looks at its shape ruled that idea out (54, 55). Not until Bancroft’s team stepped in during 2019 did researchers get a full picture, using detailed imaging, interaction experiments, and activity checks (56). Among the proteins found in Trichuris, p43 stands out for its unique sequence pattern, showing little match with known proteins outside this group. Nevertheless, when examined by Probis, certain segments resemble thrombospondin type-1 repeats, as well as regions similar to interleukin-13 receptor alpha-2 (IL-13Rα2) (57). Notably, the resemblance to IL-13Rα2 matters: this molecule binds IL-13 tightly, even at very low concentrations, preventing it from acting by serving as a non-signalling trap (57, 58).

Found by X-ray crystallography, the structure of p43 shows it is tightly packed and contains 36 cysteine residues, each forming disulfide bonds, suggesting high stability and suitability for surviving harsh digestive conditions in the gut (16). At its far end, a unique stretch rich in histidine appears, capable of binding two-charged metal ions such as zinc. Because of this trait, the protein might bind to sugar chains only when zinc is present, helping it anchor effectively. Measurements by microscale thermophoresis show that p43 binds directly to IL-13 with an apparent affinity of about 420 ± 120 nM, as determined in binding assays (16, 31). From native mass spectrometry and collision-induced dissociation, the pairing of one p43 with one IL-13 emerges clearly - these complexes shift when they grab their target. While interacting with heparan sulfate - a typical glycosaminoglycan - the bond tightens sharply, reaching roughly 10 nM strength; such a grip relies heavily on zinc being present (31).

When tested in lab settings and live organisms, p43 was found to block immune reactions triggered by IL-13. Experiments with fluid-collected abdominal cells showed that p43 sharply reduced levels of resistin-like molecule-alpha - a signal tied to IL-13 activity - yet left responses driven by IL-4 unchanged, highlighting its selective effect on IL-13 pathways (59, 60). Delivered into the airways, p43 also suppressed the IL-13-induced increase in RELM-alpha-positive lung macrophages, demonstrating its efficacy against this pathway under physiological conditions.

### 2.4 Structural features of p43/Tm-DLP-1

Looking more closely at p43 through high-resolution X-ray crystallography reveals how its 3D structure sets it apart from previously known nematode proteins. Instead of a single unit, the molecule folds into two closely connected parts, each resembling the other. A disulfide bridge holds these halves together near their point of contact. Computational models suggest this connection zone interacts with IL-13. Such repetition within the structure hints at a new evolutionary step. This may have allowed the protein to bind multiple signalling molecules (17).

Inside the p43/Tm-DPL-1 protein sits an arrangement shaped by repeated segments - one at each end - held together through a flexible middle piece. What stands out begins early: sequences resembling TSP-1 repeats are shown in red, while sequences matching IL-13Rα2 are shown in green. At one tip, a chain made entirely of histidines stretches, coloured purple here. Thirty-six cysteines twist into pairs, building bridges that shape the structure. Where interactions occur, two zones catch the eye - one yellow for IL-13 capture, another blue for glycosaminoglycan contact. Found only in certain roundworms known as Clade I nematodes (Dorylaimia), this molecule fits within the dorylipophorin group (13, 61). Looking closely at the protein surface shows several pockets, each with unique charge patterns, hinting they might interact with various small molecules. Elongated channels appear in some areas - similar to those seen in certain animal and worm proteins known for holding lipids - lined mostly with nonpolar surfaces. However, a few polar spots sit at their tips, possibly anchoring substances like retinol or fatty acids. Not all depressions share this layout; others show contrasting electrical landscapes on their exteriors, likely suited for binding chemically dissimilar partners (13) **Table 1**.

**Table 1:** The structure, secretion, and biochemical properties of P43 protein.

Aspect	Properties	Ref
Protein Name	p43 (Tm-DLP-1 in T. muris, Tt-DLP-1 in Trichuris trichiura)	
Organism	Parasitic nematodes (Trichuris spp., Clade I Dorylaimia)	
Molecular Weight	~43 kDa (p43 in T. muris), ~47 kDa (p47 in T. trichiura)	
Structural Features	Comprises internally duplicated subdomains with extensive surface-accessible cavities. Cavities exhibit diverse surface charge characteristics, suggesting binding to a range of small molecules. - Rich in histidine and cysteine, particularly in the His-rich carboxy-terminal tail (though less pronounced in some orthologs, e.g., Dioctophyme renale). Belongs to the “dorylipophorin” (DLP) family, unique to Dorylaimia nematodes. Orthologous to poly-cysteine and histidine-tailed protein (PCHTP) from Trichinella spiralis and P44 from Dioctophyme renale.	(17, 62)
Secretion Mechanism	Constitutes ~95% of proteins secreted by adult T. muris parasites. Secreted into the host intestinal environment to modulate immune responses. Likely secreted via a specialised mechanism, though specific pathways (e.g., hypodermal secretion as seen in related nematode proteins like As-p18) are not fully detailed for p43. Secretion is critical for establishing prolonged infections despite host immune responses.	(17)
Biochemical Properties	Lipid Binding: Binds fatty acids and retinol, including signalling lipids or their precursors, as demonstrated by fluorescence-based methods. Immunomodulatory Activity: Neutralises immune cytokine IL-13, contributing to immune evasion. Matrix Binding: Interacts with matrix proteoglycans, aiding in localisation or function in the host environment. - Does not show strong evidence of binding eicosanoid lipid PGE2 in fluorescence-based competition assays, though PGE2’s pH-dependent solubility might allow transient interactions with p43. - The His-rich regions may influence biochemical activity, potentially varying with the biology of different nematodes (e.g., less His-rich in D. renale).	(17, 63)
Functional Role	Facilitates chronic infections by modulating host immune responses. Likely delivers or stabilises signalling lipids to alter the host gut environment, thereby supporting anti-helminth immunity suppression. Plays a role in establishing a replicative niche for the parasite in the host intestine.	(13)
Evolutionary Context	Part of the dorylipophorin family, confined to Clade I nematodes. Evolved to support parasitic lifestyles, with structural variations (e.g., His-richness) reflecting adaptations to different host environments. Phylogenetic analysis suggests a distinct gene class within the fatty acid-binding protein family, unique to nematodes.	(13, 17)

Among known proteins, p43 stands out due to its distinct mix of amino acids - cysteine appears at 8.4%, lysine at 10.2%, proline reaches 8.8%, while histidine hits 6.1% - even when leaving aside the long histidine-heavy tail at the C-end. Stability comes sharply into focus because of 18 tightly formed internal disulfide bridges, which anchor the structure firmly. Such rigidity probably explains why digestive enzymes struggle to break it down in the gut. Longevity outside cells might stem from this same toughness; once lodged in the extracellular environment, p43 retains IL-13 for prolonged periods (13, 17). A string of around fifteen to twenty histidines at the protein’s C-terminus stands out as a notable trait. Beyond possibly binding metal ions, such stretches might influence how proteins interact, depending on acidity levels - histidine gains protons more readily when the environment turns sour. Since the space where *T. muris* lives within host cells can shift in pH, this behaviour might help regulate p43’s function (23, 64).

### 2.5 Dorylipophorin: a novel protein family

The 2025 study by Kennedy and colleagues formally designated p43 and its orthologs as members of a novel protein family termed “dorylipophorins” (Dorylaimia lipid-carrying proteins) (17). This nomenclature reflects both the phylogenetic distribution of these proteins (exclusive to the Clade I nematode order Dorylaimia) and their primary function as lipid carriers. The study demonstrated that p43 is the dominant protein in Trichuris pseudocoelomic fluid, where it likely functions as a bulk mobilizer and lipid transporter within the worms.

This finding has profound evolutionary implications. In all other studied nematode clades (III, IV, and V, collectively known as Chromadoria), the major pseudocoelomic lipid transporters are either nematode polyprotein allergens or fatty acid- and retinol-binding proteins (FARs) (19). These protein families are absent or highly reduced in Trichuris species, suggesting that dorylipophorins have functionally replaced them in Clade I nematodes. This represents an ancient functional dichotomy in nematode evolution, with distinct solutions to the fundamental physiological challenge of lipid transport evolving independently across different clades.

Importantly, the study demonstrated that the orthologue of p43 from the human whipworm *Trichuris trichiura* (designated p47, reflecting its slightly larger molecular mass) exhibits similar lipid-binding activity, including binding to fatty acids and retinol (17). Using fluorescence-based methods, both p43 and p47 were shown to bind fatty acids, including signalling lipids and their precursors, as well as retinol. This conservation of lipid-binding function across Trichuris species suggests that the dual functionality of dorylipophorins, internally as lipid carriers and externally as immunomodulators, may be a general feature of this protein family.

Based on the known molecular structure of p43, computational analysis has identified extensive surface-accessible cavities with diverse surface charge characteristics, suggesting binding of diverse small-molecule types (17). The internally duplicated subdomains are likely to exhibit divergent characteristics, potentially enabling simultaneous binding of multiple ligand types. This multi-ligand binding capacity may underlie the protein’s ability to both transport lipids internally and neutralise cytokines externally.

The evolutionary trajectory that produced dorylipophorins likely involved gene duplication and neofunctionalization of an ancestral lipid-binding protein, followed by the acquisition of IL-13-binding capability through convergent evolution of IL-13Rα2-like domains. The selective advantage conferred by IL-13 neutralisation in the mammalian intestinal niche would have driven the elaboration of this dual-function protein to its current state of extraordinary abundance in Trichuris ES products (17, 65).

## 3 Molecular mechanisms of immunomodulation

### 3.1 IL-13 binding and neutralisation

Interleukin-13 is a multifunctional cytokine belonging to the type 2 cytokine group that plays an important role in anti-helminthic immunity and cancer development. In *T. muris* infection, IL-13 plays a key role as an effector cytokine, promoting parasite expulsion by enhancing epithelial cell renewal, mucin synthesis, and smooth muscle contraction (66). Thus, it can be seen that the p43 protein’s ability to inhibit IL-13 activity directly interferes with the host organism’s most critical defence mechanism. The mode of action of the p43-IL-13 complex was elucidated by integrating structural modelling and binding studies. The region where IL-13-binding occurs is estimated to be located in the area where two duplicated p43 domains intersect, which may allow p43 to mimic IL-13Rα2 in its binding with IL-13 (16).

However, compared with the conventional decoy IL-13Rα2 receptor, whose binding affinity for IL-13 is on the picomolar scale (56), the binding affinity of p43 for IL-13 is significantly lower. Despite this, several factors underlie the enhanced efficiency of IL-13 neutralisation by p43. First, owing to its exceptionally high concentration in the intestinal microenvironment (in the micromolar range, since p43 accounts for 95% of the total ES content), p43 compensates for its relatively low affinity. Second, the anchorage of p43 to glycosaminoglycans in the extracellular matrix creates a localised IL-13-binding site that acts as an efficient IL-13 sink in response to IL-13 produced by activated Th2 cells (17, 67). Of note, p43 shows IL-13 specificity over the structurally similar IL-4, although both cytokines utilise IL-4Rα as their common receptor and share downstream pathways. Indeed, p43 specifically blocks IL-13-induced RELM-α production but does not affect IL-4-induced responses (68, 69). Likely, the differences in the molecular surfaces of IL-13 and IL-4 that bind to p43 explain the specificity of inhibition.

Effects on Tumour Biology Beyond the Mechanism of Action of p43. The biological effects resulting from IL-13 neutralisation by p43 do not end at merely blocking the IL-13-mediated pathway. IL-13 promotes M2 macrophage polarisation, which facilitates tumour progression by promoting angiogenesis, inhibiting T cell cytotoxicity, and producing anti-inflammatory cytokines (70). Neutralising IL-13 via p43 may shift macrophages from M2 to M1 polarisation, boosting tumour immune surveillance. Moreover, IL-13 inhibits CD8+ T cell-mediated cytotoxic responses and promotes Treg-mediated suppression; p43-based inhibition may help reverse these effects (71).

### 3.2 GAG-mediated tethering mechanism

In **Figure 1**, what makes p43 stand out in immune regulation is how it attaches to sugar chains called glycosaminoglycans within body tissues. Because of this link, p43 builds up near gut lining cells - its main workplace - and stays longer by avoiding quick removal, while possibly aligning just right to block IL-13 (16, 72). When tested with heparan sulfate - a common lab version of these sugars - the grip p43 form is extremely strong, around 10 nanomolar, far tighter than its bond with IL-13 itself (13, 16). That contrast in stickiness matters biologically. Strong anchoring means that even when p43 repeatedly grabs and releases IL-13, it does not float away from its target.

**Figure 3:**
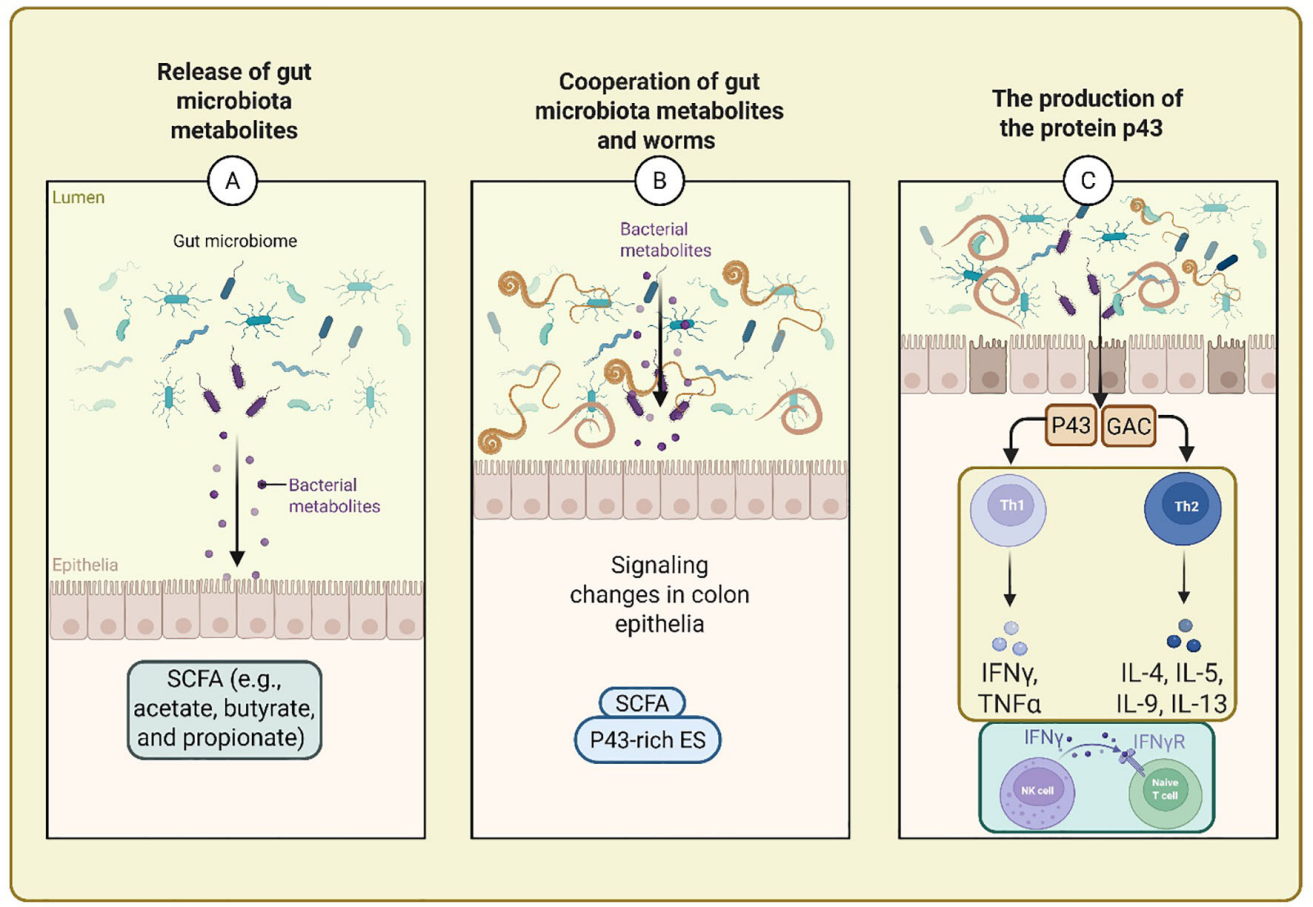
Modulation of the immune response. The biosynthesis of protein p43 in adult and fully developed worms demonstrates a close association with its interaction with glycosaminoglycans and the cytokine IL-13, which plays a pivotal role in this biological process. An adult worm is revealed, its anterior aspect securely anchored within the epithelial plane, while its posterior region, notably rounded, remains freely suspended within the internal channel of the formation. CD4+ T helper cells, by consistently secreting interleukin-10, adeptly modulate the activity of interferon-gamma (IFN-”γ”), a principal factor in the host’s defence against chronic infections, thereby ensuring a stable immune environment. Mature nematodes produce a significant quantity of p43, which resides in their ambient surroundings and adheres, for instance, to the extracellular matrix. The successive infections resulting from *T. muris* invasion of L1 larvae trigger a Th2 immune response and upregulate IL-13 production. Subsequently, GAG-bound p43, with high binding affinity, sequesters IL-13, thereby inhibiting its functional activity. This action leads to a decelerated and compromised immune defence in the host, which may consequently augment the parasite’s survival likelihood. This figure was generated using BioRender software, version 04.

When IL-13 enters the mix with p43 already attached to heparan sulfate, surface plasmon resonance shows that something unusual happens: instead of staying put, p43 lets go of the GAG because it now binds to IL-13 (16). What unfolds next looks like a handoff: once p43 grabs IL-13, its shape shifts just enough to break free from the sugar chain. Because of this shift, the duo floats away, clearing IL-13 from nearby spaces while freeing up the GAG spot for another p43 molecule to take hold. In whipworm infections, the long-term presence of the parasite reshapes the tissue landscape in the cecum, marked by increased levels of heparan sulfate, fibronectin, and collagen I (73). As these structural changes build out the extracellular framework, they likely provide more docking sites for p43, boosting the amount of IL-13 captured locally as immune activity ramps up **Figure 3**.

When zinc ions are present, p43 binds more strongly to GAGs; without them, binding weakens sharply due to chelation. This reliance on zinc lacks a full explanation, though hints point to links forming between p43’s string of histidines and sulphated parts of GAGs. In adult *T. muris* excretory-secretory material, zinc appears at higher concentrations. One possibility - though not confirmed - is that the worm adjusts zinc levels nearby to favour these molecular attachments (13, 60, 72).

### 3.3 Lipid carrier function

Found inside cells, p43 now appears to act like a dorylipophorin - a clue that deepens its role in immune regulation. Carrying fats through the body, it attaches to fatty acids and retinol - molecules involved in cellular communication (17). Because of this ability, changes in immunity might follow several different routes.

The signalling functions of fatty acids help control immune activity. Prostaglandins and leukotrienes are derived from certain polyunsaturated fatty acids; these compounds influence the course of inflammation. When p43 binds distinct fatty acids, the local mix of lipid-based signals might shift, possibly reducing eicosanoids that drive inflammation rather than just supporting reactions: Retinol and its metabolite, retinoic acid, shape how immune cells mature and protect mucosal surfaces. Because p43 can bind retinol, it may reshape what dendritic cells do and how T cells respond (74). Looking closely at p43’s form shows several open pockets on its surface - spaces capable of fitting various lipids (61, 75). Pockets lined mostly with nonpolar residues tend to host fatty acid tails, whereas ones with ionic linings might attract phospholipid heads or retinol OH moieties. Because lipid-affine zones vary, p43 likely shuttles several lipid types at once - each playing a role in immune tuning. Although distinct chemically, these regions together support concurrent cargo handling **Table 2**.

**Table 2:** Molecular mechanisms of p43 immunomodulation.

Topic	Mechanism & key findings	Molecular/structural basis	Functional consequences	Ref
The p43/Tm-DLP-1 Protein	It is the dominant protein in T. muris excretory/secretory (E/S) products, accounting for over 90% of the total secreted protein	. P43 is composed of 397 amino acids, with 36 cysteine residues (9%) and a histidine-rich C-terminal region (45% of the terminal 29 residues are His). Its structure includes subdomain homology to thrombospondin type 1 and the IL-13 receptor α2.	Dominates the secretome, highlighting its crucial role in host-parasite interaction and immune evasion.	(13, 16, 57)
IL-13 Binding & Neutralization	p43 binds directly to IL-13, the key effector cytokine for worm expulsion, and neutralises its function both in vitro and in vivo.	Direct binding was demonstrated via co-immunoprecipitation and native mass spectrometry, showing a tight 1:1 complex.	Inhibits IL-13-dependent gene expression (e.g., Muc5ac), blocking the host’s protective Th2 immune response and allowing chronic infection.	(13, 16, 57)
GAG-Mediated Tethering	p43 binds glycosaminoglycans (GAGs), particularly heparan sulfate (HS), enabling it to tether to the host colon’s extracellular matrix (ECM).	The interaction with HS and IL-13 appears to compete for the same or overlapping binding sites on the p43 molecule, creating an “on-off” switch mechanism.	Tethering provides a bound, localised reservoir of p43, ready to intercept IL-13 near the epithelial surface, where the parasite resides.	(30)
Lipid Carrier Function	P43 binds fatty acids and retinol, including signalling lipids and their precursors, establishing it as a lipid carrier.	Fluorescence-based methods demonstrated lipid-binding. Structural analysis reveals extensive surface-accessible cavities with diverse charge characteristics that bind multiple small-molecule types.	This activity may allow the parasite to manipulate the host environment by delivering or sequestering key signalling lipids at the infection site.	(13, 16, 57)
Zinc-Dependent Interactions	The binding of p43 to heparan sulfate (HS) is highly dependent on the presence of zinc ions, with minimal binding observed in its absence.	The histidine-rich C-terminal tail of p43 is hypothesised to mediate this Zn²^+^-dependent GAG binding, though the exact mechanism is still under investigation.	Zinc-dependent tethering suggests that the local metal ion concentration in the gut could regulate p43’s localisation and, consequently, its IL-13 neutralising activity.	(13, 16, 57)
Effects on Immune Cell Populations	p43 inhibits the development of IL-13-induced RELM-α^+^ alternatively activated macrophages in vivo in the lung.	It also inhibits IL-13- and IL-4-dependent gene expression in murine macrophages, such as RELM-α and Muc5ac.	Directly alters macrophage phenotype and function, demonstrating the protein’s ability to shape the immune landscape beyond simple cytokine neutralisation.	(13, 16, 57)

One role of p43, the main pseudocoelomic protein, may mirror that of serum albumin in vertebrates or lipophorin in insects - shuttling fats across tissues inside the nematode (61, 76). Repurposing this fat transporter for outside immune influence shows how evolution adapts existing tools: a molecule built for inner tasks now shapes host responses instead.

### 3.4 Zinc-dependent interactions

**Figure 4:**
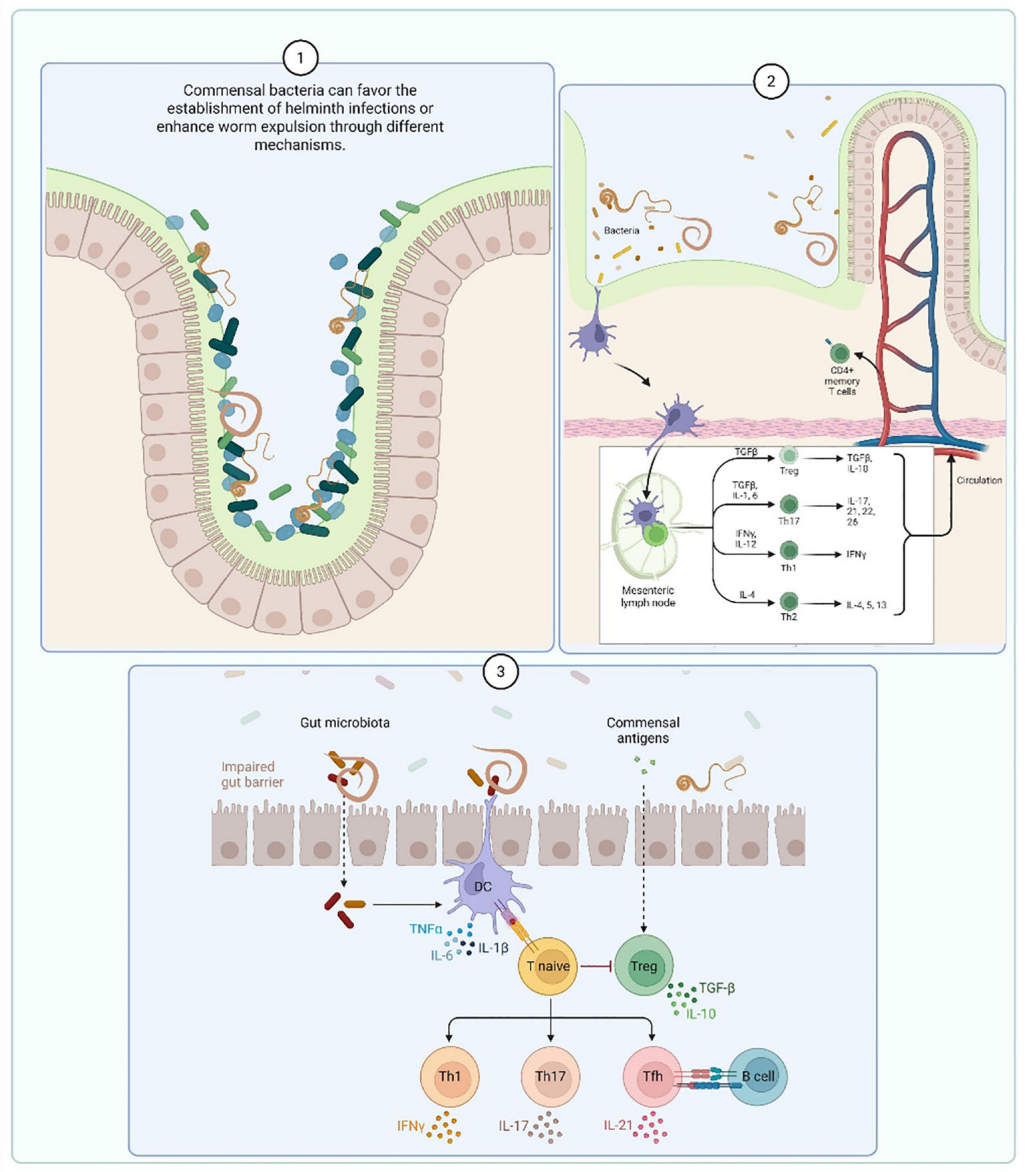
The intricate relationship between the microbiota and helminth infections reveals a fascinating interplay that influences both the establishment and expulsion of these parasitic worms. Helminth infections can be either promoted or hindered by commensal bacteria through distinct mechanisms. For instance, some helminths require the host’s microbiota to establish infection and to develop their own microbiota within the host’s gut, which is colonised by bacteria from the host. This relationship often leads to a more diverse gut microbiota, which can lower the risk of certain diseases by regulating immune responses. The presence of specific bacterial taxa can influence a host’s susceptibility to parasitic infections, in part by expanding the regulatory T cell (Treg) population, which plays a significant role in immune regulation. Although helminths are typically considered harmful, commensal bacteria can help regulate these infections, assisting in their control and revealing a complex symbiotic relationship that highlights the potential therapeutic implications of manipulating the microbiota to manage helminth infections effectively. This figure was generated using BioRender software, version 04.

As shown in **Figure 4**, one reason the p43-GAG interaction relies on zinc is that metal ions fine-tune p43’s immune activity. Essential though it is for many bodily functions - immune cells included - zinc shows strong effects when present in too little or too much (77). Beyond diet, gut microbes help control zinc balance, even shifting blood concentrations during parasitic worm infections (78). Sitting at one end of the protein, the cluster of histidines in p43 could easily grab onto zinc, since such amino acids often bind metals inside biomolecules. A stretch of 15 to 20 histidine residues in a row forms a strong binding site for metal attachment, possibly leading to shape shifts dependent on zinc and influencing how GAGs and IL-13 bind. Early data indicate that when zinc binds, parts of p43 might reorganise, revealing or reinforcing the area where GAGs bind.

Surprisingly, p43, zinc, and gut microbes might form a self-adjusting system. Depending on how much zinc is present, different bacteria dominate the microbial community - some grab it better than others (79, 80). When worms shift, which microbes live in the gut, they may indirectly affect the amount of free zinc in the gut, thereby altering how strongly p43 binds to GAGs. Far from straightforward, this link among a parasite’s protein, the body’s zinc balance, and resident microbes opens an unexplored path forward.

### 3.5 Effects on immune cell populations

Beyond direct IL-13 neutralisation, p43 and other *T. muris* ES components modulate multiple immune cell populations, creating a favourable environment for parasite persistence. These immunomodulatory effects have direct relevance to cancer therapy, as many of the same immune cell populations targeted by helminth products are also critical regulators of anti-tumour immunity.

#### 3.5.1 Dendritic cells

*T. muris* ES products modulate dendritic cell maturation and function, promoting a tolerogenic phenotype characterised by reduced expression of co-stimulatory molecules and increased production of IL-10 (81). Laan and colleagues demonstrated that T. suis ES contains prostaglandin E2 (PGE2), which downregulates inflammatory cytokine production from lipopolysaccharide-stimulated human dendritic cells (82, 83). These dendritic cell modifications suppress Th1 and Th17 responses while promoting the induction of regulatory T cells.

#### 3.5.2 Macrophages

IL-13 neutralisation by p43 directly affects macrophage polarisation. IL-13 is the canonical driver of M2 macrophage polarisation, promoting the expression of arginase-1, the mannose receptor, and anti-inflammatory cytokines (70). By sequestering IL-13, p43 may promote M1 polarisation, characterised by enhanced antigen presentation, production of pro-inflammatory cytokines (TNF-alpha, IL-1beta, IL-12), and direct cytotoxicity against tumour cells. This M1/M2 repolarisation could significantly enhance anti-tumour immune responses.

#### 3.5.3 T cells

Chronic *T. muris* infection induces a regulated Th1 response with significant IL-10 production, which suppresses effector T cell function (84, 85). However, p43 itself appears poorly immunogenic during natural infection, with limited antibody response and no discernible T cell response even in chronic infection (17, 67). This lack of immunogenicity may reflect compartmentalisation of p43 to the intestinal niche, where it remains sequestered from antigen-presenting cells. The immunological invisibility of p43 during infection suggests that therapeutic administration might not elicit neutralising immune responses, a favourable property for biologic drugs.

#### 3.5.4 Regulatory T cells

*T. muris* infection robustly induces regulatory T cells (Tregs), which produce IL-10 and TGF-beta to suppress inflammatory responses. While Treg induction is beneficial for parasite survival, excessive Treg activity in the tumour microenvironment suppresses anti-tumour immunity. The net effect of p43 on Treg function is complex: IL-13 neutralisation may reduce Treg suppressive capacity, while other ES components may independently promote Treg induction (86, 87).

#### 3.5.5 Innate lymphoid cells

Type 2 innate lymphoid cells (ILC2s) are early producers of IL-13 in response to helminth infection, and their activation is modulated by ES products (88). The ability of p43 to scavenge IL-13 may create a negative feedback loop that limits ILC2 activation, potentially reducing the overall magnitude of the type 2 immune response **Figure 4**.

## 4 Nematode-microbiota interactions

### 4.1 The gut microbiota landscape

Among mammals, the digestive system hosts a varied mix of microbes - bacteria, archaea, fungi, and viruses - together named the gut microbiota. Home to some 10^13–10^14 individual cells across more than a thousand types, this living network helps break down nutrients, shape immune responses, and block harmful invaders (89, 90). While its balance naturally shifts, research now links disruptions in these communities to conditions such as inflammatory bowel disease, metabolic issues, autoimmunity, and tumours (91, 92).

Though hidden within the digestive tract, tiny organisms shape how tumours grow, spread, and react to treatment. These microbes break down plant fibres, releasing molecules - like SCFAs - that guide immune activity and alter cancer cell behaviour (93, 94). One such molecule, butyrate, fuels healthy colon cells while blocking enzymes in malignant cells, slowing their division and triggering self-destruction; it also promotes specialised immune regulators (88, 91, 92). Meanwhile, propionate and acetate bind to surface sensors on immune cells - specifically GPR43 and GPR41 - and fine-tune inflammation levels across tissues (95–97).

Some bacteria are linked to a higher chance of developing colon tumours. Fusobacterium nucleatum can promote tumour growth by activating beta-catenin signalling while weakening immune responses against cancer cells (98). On the flip side, *Faecalibacterium prausnitzii*, along with *Akkermansia muciniphila*, may lower such risks. Their benefit might come from releasing substances that calm inflammation and support gut lining strength (99) **Table 3**.

**Table 3:** Mechanism of interaction of nematode and bacteria.

Nematode type	Bacteria involved	Interaction type	Mechanism	Refs
Entomopathogenic Nematodes (e.g., Heterorhabditis bacteriophora, Steinernema spp.)	Photorhabdus luminescens, Xenorhabdus spp.	Obligate Mutualism	Nematodes carry symbiotic bacteria in their gut, releasing them into insect hosts to produce toxins (e.g., Tca, mcf) and enzymes that kill the host and provide nutrients for both partners. Bacteria also produce antibiotics to reduce competition from other microbes.	(100)
Trichuris spp. (Whipworms)	Host Gut Microbiota	Indirect Interaction	Secreted proteins such as P43 modulate host immune responses (e.g., IL-13 neutralisation), potentially altering gut microbial composition. Specific bacterial interactions are not detailed, but AMPs from other nematodes suggest a role in modulating the microbiome.	(17)
Caenorhabditis elegans	Pseudomonas aeruginosa (with Pf4 phage)	Pathogenic/Modulated by Phage	Pf4 phage suppresses bacterial quorum sensing and pyocyanin production, reducing C. elegans immune detection and enhancing bacterial survival.	(101, 102)
Caenorhabditis elegans	Native Microbiome (e.g., Enterobacteriaceae, Pseudomonas, Ochrobactrum)	Commensal/Neutral	Native bacteria colonise the nematode gut, influencing development and immunity without causing harm. Specific strains, such as Ochrobactrum MYb71, persist in the gut, suggesting a stable interaction.	(103)

### 4.2 Trichuris-microbiota crosstalk

Strong data show Trichuris species do not act alone - they shape, and are shaped by, gut microbes in ways that shift how infections progress and how the body responds immunologically. At play here: touch-based links between worm and bacteria, chemical signals passed back and forth, and changes driven by the host’s altered defences (62, 104). In cancer settings, a three-way dynamic emerges among parasite, resident microbes, and immune cells. Visually, this network shows round-trip influences across all parties. Molecules released by the worm - like p43/Tm-DLP-1, tiny delivery bubbles called vesicles, and small biochemicals - affect microbial balance alongside immune behaviour. Immune reactions in the host shift under the sway of microbial byproducts, such as SCFAs. By shaping the tumour microenvironment, these substances boost defences against cancer while quieting harmful inflammation. Balance often returns where immunity had gone awry (41, 60).

Work led by White showed that egg hatching in *T. muris* fails in germ-free mice, suggesting that bacteria are essential triggers (47, 105). Later research revealed that certain microbes, especially Escherichia coli and related Enterobacteriaceae, can kickstart emergence through molecular structures and enzyme functions (28, 106, 107). Far from accidental, this reliance on gut microbes for development reflects deep evolutionary shaping; it ties the worm’s survival tightly to host-associated bacterial networks. In addition to egg-hatching needs, *T. muris* infection reshapes the gut microbial community. In C57BL/6 mice, long-term infection sharply lowers Bacteroidetes variety and numbers, while Firmicutes and Proteobacteria rise in parallel (27, 108). Such changes in bacterial makeup are linked to disrupted metabolism - especially weaker breakdown of dietary fibre and lower short-chain fatty acid output. This imbalance might indirectly aid the parasite: fewer competing microbes mean easier access to nutrients and a more suitable intestinal environment.

Surprisingly, adult *T. muris* worms harbour a unique set of microbes distinct from those in the host’s gut lumen (47). Because these parasites cannot genetically produce certain compounds - like butyrate - detected in their excretory-secretory material, microbial partners probably shape the chemical output (11, 109). Rather than acting alone, the worm’s resident bacteria might serve as a hidden metabolic helper, broadening what the organism can chemically achieve.

### 4.3 Metabolite-mediated interactions

One study found major shifts in gut chemicals during long-term *T. muris* infection. By day 41, researchers saw higher levels of certain amino acids - like phenylalanine, serine, and leucine - in stool samples; meanwhile, compounds tied to vitamin D, fats, and glycerophospholipids dropped sharply (108). Though host cells, microbes, and the worm itself likely all play roles, the extent to which each contributes remains unclear. More amino acids might mean the body breaks down more proteins during tissue repair. Lower levels of fat-related molecules may stem from disrupted bacterial activity due to an imbalance in the gut microbiota (110–112). Scientists continue examining which factors drive these chemical shifts most strongly (113).

Among the compounds found in ES-enriched fractions from *T. muris*, several display known abilities to influence immune responses. Starting with glucosamine - a piece of both microbial walls and host molecules - it acts against inflammation by blocking NF-kappaB pathways (108). Then there is uridine, typically part of RNA synthesis, which modulates immune cell behaviour via purinergic signalling. Also at play is butyrate; its origin probably lies with microbes living alongside the worm, not the parasite directly - yet it strongly shapes immunity, boosting regulatory T cells while quieting pro-inflammatory signals (110–112). Metabolites such as succinate and lactic acid found in *T. muris* ES fractions might originate from low-oxygen processes within the worm’s gut or its microbial partners (16, 45). Although distinct in their actions, each plays a role in shaping immune responses. Succinate binds to SUCNR1 on dendritic cells, increasing IL-1beta output. Meanwhile, lactic acid alters T cell behaviour - dampening their activity while encouraging the rise of regulatory T cells, especially in tumours (113).

### 4.4 Short-chain fatty acids and immune regulation

Found in the gut, short-chain fatty acids - like acetate, propionate, and butyrate - help shape how microbes talk to their host. Because of this role, they influence what happens during worm infections and when bacteria break down dietary fibre; these compounds affect immunity in various ways (114–116).

Though often overlooked, butyrate stands out because it fuels colon cells and also guides gene expression. By blocking HDAC enzymes, it loosens DNA packaging near gene switches, turning up protective genes like p21 and p53, yet quieting those involved in inflammation (105). Oddly enough, in tumours, its actions split - feeding healthy gut lining cells even as it triggers self-destruction in malignant ones. This contrast appears to be tied to faulty metabolism in cancer cells; their reliance on sugar rather than oxygen disrupts the proper breakdown of butyrate (117).

Later in *T. muris* infection, levels of butyrate in the gut lumen drop, while host cells reduce the expression of transporters that transport butyrate, suggesting a shift toward alternative fuel sources (23, 118). With less butyrate available, inflammation during early infection could worsen. Still, traces found in excretory-secretory materials might calm immune activity right where the parasite meets tissue. Immune cells respond to SCFAs when these molecules trigger G-protein-coupled receptors. Though neutrophils and macrophages quell inflammation via acetate and propionate acting on GPR43 (FFAR2), dendritic behaviour shifts under GPR41 (FFAR3) signals, influencing T cell development (119, 120). Butyrate activates GPR109A (HCAR2) in certain immune cells, boosting the production of IL-10 and IL-18 - both calming cytokines - and supporting the growth of regulatory T cells (121). With *T. muris* infection shaping immunity, such changes driven by SCFAs might fit within that broader shift.

### 4.5 Implications for cancer therapy

Though tiny, roundworms influence gut microbes and immune responses in ways relevant to treating tumours. A new view now taking shape points to coordinated changes across all three elements - host defences included - as a route toward stronger treatment outcomes. Effects may stack when these components interact under medical guidance. This approach leans on overlapping biological pathways rather than isolated actions (122).

Delivered straight into the gut lining, p43/Tm-DLP-1 might block IL-13 right inside tumours - especially useful in colorectal cases where the intestinal space is reachable - avoiding broad immune suppression. Thanks to its ability to bind sugar chains, the molecule tends to remain in the intestinal tissue layers, possibly reducing unintended effects elsewhere (72). Another path opens when parasitic worm secretions - like p43, tiny particle carriers, and metabolic byproducts - are paired with treatments that reshape microbial balance. Instead of acting alone, these elements may work better alongside bacterial helpers; for instance, introducing microbes such as *Faecalibacterium prausnitzii* or *Roseburia* species could team up with p43 to boost short-chain fatty acid signalling while simultaneously quieting IL-13 (5, 122).

It might start with gut balance - helminth substances possibly correcting microbial imbalance seen often in people fighting cancer, especially after chemo, antibiotic use, or because of the disease. Because these treatments disturb natural bacteria, introducing worm-derived molecules may help good microbes thrive again, creating conditions that boost the effectiveness of immunotherapy drugs, since those responses rely heavily on the right gut environment (123, 124). Another angle: chemicals released by *T. muris*, such as short-chain fatty acids, protein building blocks, and components of genetic material, could directly interfere with tumour metabolism. Since cancer cells consume energy differently from healthy cells, supplying specific small molecules via excretory-secretory products might slow malignant growth without harming normal cells (125).

## 5 Cancer biology and IL-13 signalling

### 5.1 IL-13 in tumour development

Appearing in various stages of cancer development, interleukin-13 influences how tumours start, grow, spread, and resist treatment. While some effects occur directly within cancer cells, others arise from alterations in surrounding tissue conditions (126).

One way IL-13 acts directly on tumour cells is by binding to the IL-13 receptor complex. Though healthy cells usually carry the IL-13Rα1/IL-4Rα pair - known as the type II IL-13 receptor - a large number of malignant cells instead express IL-13Rα2. Once thought to be a receptor that soaks up IL-13 without signalling, IL-13Rα2 can actually transmit signals in specific contexts (127). When IL-13 binds to these receptors, it activates the JAK-STAT pathway, particularly by phosphorylating STAT6. This shift involves genes that support tumour growth, spread, and resistance to death. Epithelial-mesenchymal transition is driven by IL-13 in colorectal cancer, setting the stage for tumour spread. Instead of simply adding forces, IL-13 shifts the balance - fewer epithelial signals like E-cadherin appear while vimentin, N-cadherin, Snail, and Slug rise in response. Movement and invasion grow stronger under this shift, driven not by chance but by defined molecular changes. Behind it all, STAT6 steers gene activity, linking directly to how TGF-beta pathways behave in tandem (128).

**Figure 5:**
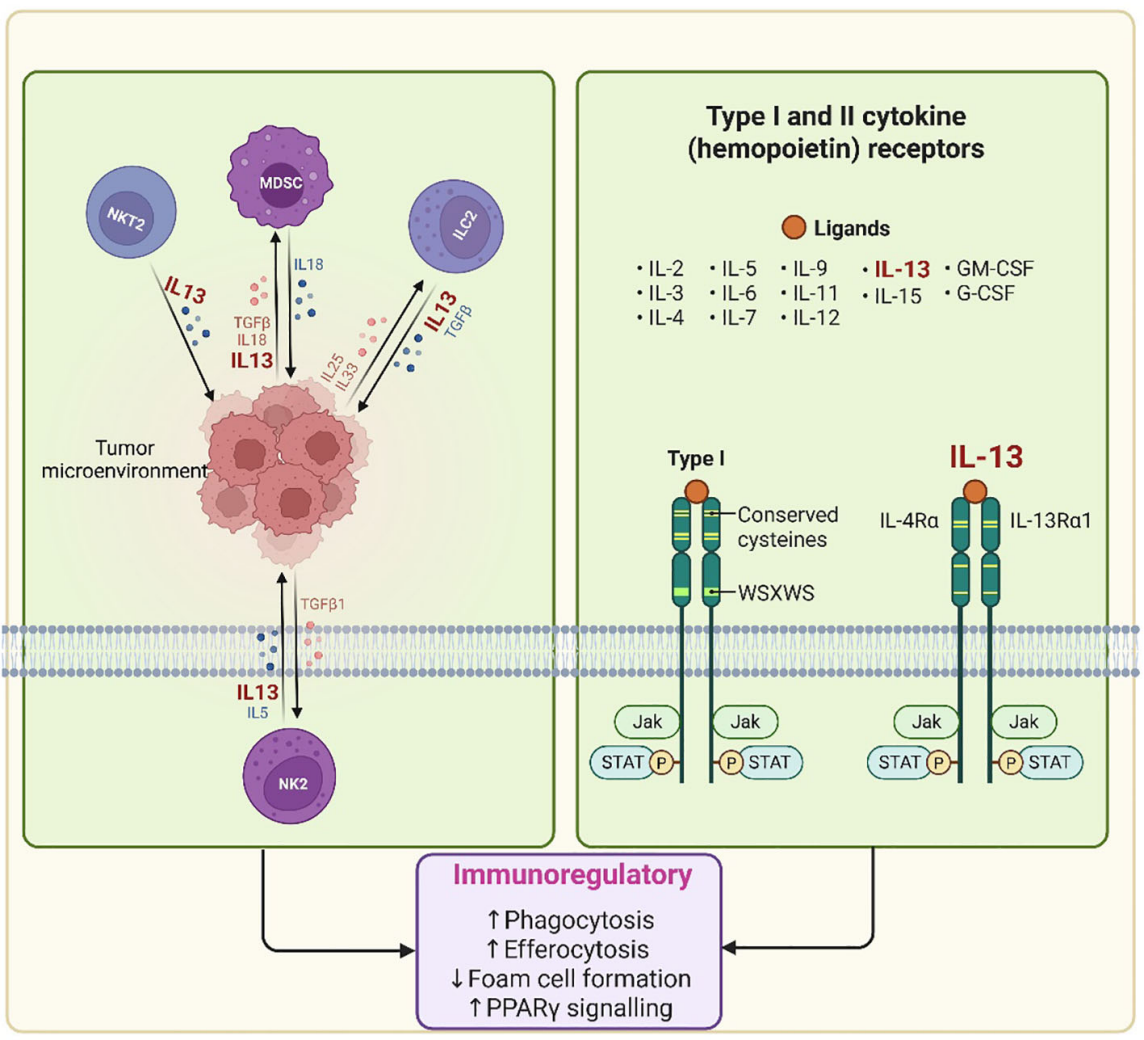
Detailed description of IL-13 and IL-4 receptors. There are two distinct receptors for IL-13:IL-13Rα1 and IL-13Rα2. To perform its physiological function, IL-13 must first bind another protein component to form a heterodimeric receptor, and signalling occurs only then. The functional heterodimer consists of IL-4Rα and IL-13Rα1. This is called the type II IL-4 receptor because it can also bind IL-4. A different combination can be formed when IL-4Rα binds to γc, forming the type I IL-4 receptor and responding to the IL-4 signalling pathway alone. On the other hand, IL-13Rα2, which is highly specific for IL-13, does not induce IL-13–target gene expression but rather functions as a decoy receptor, binding and sequestering IL-13. This figure was generated using BioRender software, version 04.

IL-13 enhances chemoresistance, thereby reducing the effectiveness of cancer therapy. Pretreating colorectal cancer cells with IL-13 lowers their response to 5-fluorouracil and oxaliplatin - this shift comes alongside higher levels of protective proteins like BCL-2, BCL-XL, and MCL-1 (20, 129). Because STAT6 drives genes that block cell death, chemo loses some of its punch, suggesting that blocking IL-13 might restore its effectiveness. In breast tumours, lung metastasis becomes more likely when IL-13 is active; pancreatic cancers rely on it to build a dense tissue around them; Hodgkin lymphoma even uses self-made IL-13 to fuel unchecked growth (130). Given its broad influence across cancers, targeting IL-13 signalling emerges as a promising path forward **Figure 5**.

### 5.2 IL-13-mediated immune evasion

Immune defences struggle in the area around tumours, where conditions become harsh. Here, IL-13 acts as a key architect, shaping lasting resistance against attacks. By shaping behaviour across various immune cells, it builds hidden pathways that allow cancer to evade detection and destruction (131). Suppressing immune responses against tumours is a key role of regulatory T cells (Tregs), and interleukin-13 enhances their ability to do so. Within the tumour area, IL-13 supports both the survival and growth of Tregs; at the same time, these cells produce more IL-13, reinforcing an escalating cycle of suppression (132). Dendritic cells altered by IL-13 help transform regular CD4+ T cells into new regulatory T cells, thereby indirectly expanding their numbers.

Appearing early in tumour settings, myeloid-derived suppressor cells significantly dampen immune responses. Because of IL-13 signalling, these cells gather more densely while enhancing their ability to block immunity. Triggered by IL-13, they start generating arginase-1 - an enzyme that reduces available L-arginine levels. With less L-arginine available, T cells struggle to multiply or function effectively (133). Alongside this metabolic disruption, MDSCs produce reactive oxygen species. Nitric oxide joins them, together weakening T cell killing capacity through direct interference. One way IL-13 helps tumours evade immune surveillance is by shifting macrophage behaviour. Shaped by IL-13, these cells turn into M2 types - marked by CD206, arginase-1, and calming signalling molecules (70, 134). Often found in solid cancers, tumour-linked macrophages act like M2 variants, fuelling spread, blood vessel formation, and tissue invasion - all while quieting T cell attacks. Much of this shift is driven by the IL-13/STAT6 pathway, which guides macrophages toward a supportive role. Blocking IL-13 could redirect their function, altering their behaviour within tumours.

CD8+ cytotoxic T cells drive the body’s defence against tumours; meanwhile, IL-13 interferes with their activity. When exposed to IL-13, these T cells produce less IFN-gamma and TNF-alpha, exhibit slower release of toxic granules, and enter exhaustion more readily (22, 135). Blocking IL-13 could shift the balance - p43 might revive CD8+ T cell attack strength, increasing the destruction of cancer cells.

### 5.3 Therapeutic targeting of IL-13

Focus on IL-13 in tumour processes has driven efforts to block it or its receptor pathways. Though effective in asthma, lebrikizumab - a high-affinity binder of IL-13 - now draws interest for treating fibrosis (136). Because it is sizable, moving it into dense tissues becomes difficult; delivering it throughout the body may also weaken immune responses. Targeting IL-13Rα1, aminralimab works differently from agents aimed at the cytokine itself. Meanwhile, tralokinumab, used for skin inflammation, struggles in cancer settings - its structure can trigger unwanted reactions and widespread biological impact (136, 137). An engineered toxin targeting IL-13Rα2, cilengitide, adds another route to the list under study (138).

Unlike current IL-13 blockers, p43 stands apart in key ways. At just 43 kDa, it is much more compact than antibody forms that weigh in at 150 kDa - this size difference may allow deeper access into tissues like the gut lining, where colon cancers arise. Because it naturally binds to glycosaminoglycans, it tends to stay put near the site of delivery, building up locally while sparing the rest of the body from wide dispersion. Having evolved alongside mammalian immunity over aeons, it could be integrated into biological systems, leading to fewer immune reactions than synthetic alternatives. What makes p43 stand out is its ability to bind both IL-13 and GAGs at once - a trait absent in current IL-13 treatments. Because it binds to GAGs, p43 can remain in place long enough to capture freshly released IL-13 molecules, reducing the frequency of administration. Though other inhibitors drift away, these hold position, offering longer-lasting interference right where needed (136, 137) **Table 4**.

**Table 4:** Comparison of IL-13 inhibitors for cancer therapy applications.

Property	p43/Tm-DLP-1	Anti-IL-13 mAbs	IL-13Rα2 antagonists
Molecular weight	~43 kDa	~150 kDa	Variable
IL-13 binding affinity	~420 nM	~1 pM	Variable
GAG tethering	Yes (Kd ~10 nM)	No	No
Tissue penetration	Good	Limited	Variable
Local retention	High	Low	Low
Additional functions	Lipid carrier	None	None
Immunogenicity	Low (natural protein)	Moderate	Variable
Production system	Recombinant/biochemical	CHO cells	Variable
Development stage	Preclinical	Phase II-III	Phase I-II

## 6 Animal trials and preclinical evidence

### 6.1 Colorectal cancer models

Colorectal cancer represents the most extensively studied malignancy in the context of helminth-derived immunotherapy, reflecting both the intestinal tropism of Trichuris species and the strong mechanistic rationale for IL-13 inhibition in this cancer type. Multiple preclinical models have demonstrated anti-tumour effects of helminth products, although direct studies with purified p43 in cancer models remain limited (139).

The azoxymethane/dextran sodium sulfate (AOM/DSS) model of colitis-associated colorectal cancer has been particularly informative. In this model, administration of *Taenia crassiceps* excreted-secretory products (TcES) significantly inhibited colonic tumour formation, with 45% of treated mice remaining tumour-free and the remaining 55% developing smaller tumours than untreated controls (140, 141). Mechanistic analysis revealed that TcES reduced STAT3 phosphorylation by downregulating the IL-6 receptor, inhibited nuclear translocation of beta-catenin, and decreased expression of the proto-oncogene Cyclin D1 (142).

Studies with *Echinococcus granulosus* hydatid cyst fluid (HCF) in heterotopic syngeneic mouse models of colorectal cancer demonstrated significant anti-tumour effects. BALB/c mice transplanted with CT26 colorectal cancer cells showed a 40% reduction in tumour incidence when immunised with HCF before tumour challenge, and 40% increased survival when HCF was administered after tumour establishment (143, 144). These effects correlated with increased serum TNF-alpha and IFN-gamma levels, suggesting immune-mediated tumour suppression.

Anti-HCF antibodies generated in rabbits cross-reacted with heat shock protein 70 (HSP70) from *E. granulosus*, which shares 60% homology with mortalin. This protein binds p53 and inhibits its anti-proliferative activity (143). This cross-reactivity raises the possibility that helminth-derived antibodies may contribute to anti-tumour effects by targeting host proteins involved in tumour suppression. *T. muris* ES products and specifically p43 have been primarily studied in the context of anti-helminth immunity rather than cancer models. However, the demonstrated IL-13-inhibitory activity of p43 is directly relevant to colorectal cancer, where IL-13 promotes tumour progression. Bancroft and colleagues showed that p43 inhibits IL-13 function both *in vitro* and *in vivo*, reducing IL-13-induced RELM-alpha production and STAT6 phosphorylation (16). These effects are predicted to suppress IL-13-driven tumour progression, although direct cancer model studies with p43 have not yet been conducted.

### 6.2 Hepatocellular carcinoma models

Hepatocellular carcinoma (HCC) is another malignancy in which IL-13 signalling promotes tumour progression, making it a potential target for p43-mediated therapy. The liver is exposed to intestinal-derived antigens and metabolites through the portal circulation, creating an immunological environment influenced by gut helminth infections (145–147).

Studies with T. spiralis excretory-secretory products in H22 hepatocellular carcinoma-bearing mice demonstrated significant anti-tumour activity. ESP administration inhibited tumour growth, promoted mitochondrial apoptosis in tumour cells, and enhanced Th1 cytokine responses (IFN-gamma and TNF-alpha) (148, 149). The anti-tumour effects were attributed to both direct cytotoxicity against tumour cells and indirect immune modulation through enhanced Th1 responses. IL-13 levels are elevated in human HCC and correlate with poor prognosis (150, 151). IL-13 promotes HCC progression by activating STAT6 signalling, upregulating anti-apoptotic proteins, and suppressing anti-tumour immune responses. The ability of p43 to neutralise IL-13 suggests potential applicability in HCC, although liver delivery would require parenteral administration rather than the oral route used for intestinal cancers.

### 6.3 Other cancer types

Beyond gastrointestinal cancers, helminth-derived products have shown anti-tumour activity in models of breast cancer, melanoma, osteosarcoma, and lung cancer (152). Diverse mechanisms, including direct cytotoxicity, modulation of immune cells, and anti-angiogenic activity, mediate these effects.

In breast cancer models, Toxoplasma gondii-derived antigens and recombinant proteins have demonstrated anti-tumour effects by inducing CD8+ T cell responses and downregulating metastasis-promoting receptors, including CXCR4. While these studies used protozoan rather than nematode products, the shared principle of pathogen-derived immune stimulation applies across parasitic taxa (153, 154). Trichinella spiralis-derived TPD52 (tumour protein D52) induced apoptosis in osteosarcoma cells both *in vitro* and *in vivo*, and anti-TPD52 antiserum increased serum levels of TNF-alpha, IL-12, and IFN-gamma in tumour-bearing nude mice. This study demonstrated that helminth-derived molecules can trigger anti-tumour immune responses even in the absence of adaptive immunity, suggesting innate immune mechanisms of tumour suppression (155). Lung cancer models have also shown susceptibility to helminth product-mediated therapy. T. spiralis muscle larva ES products induced apoptosis in H446 lung cancer cells through the mitochondrial pathway, upregulating pro-apoptotic genes (Bax, Cyt-C, Apaf-1, caspase-9, caspase-3) while downregulating anti-apoptotic BCL-2 and Livin (156, 157).

### 6.4 Mechanistic insights from animal studies

Though animal research varies widely, patterns emerge when examining how molecules from parasitic worms influence tumour growth (158). Such insights help anticipate how p43/Tm-DLP-1 might behave in similar settings, guiding later experiments. Evidence gathered from lab and animal tests using worm-based compounds in cancer contexts is outlined below. In Panel A, separate investigations involving different cancers are displayed - each listing the specific worm substance used, the testing framework, along with the central outcomes observed. What happens in animals shows up here: immune cells shift, cytokines respond, signals change, and the tumour microenvironment adapts. Moving forward, step by step unfolds: early lab work leads into human testing, progressing across trial stages (158).

Immune cell modulation shifts when exposed to helminth-derived substances: CD8+ T cells increase their ability to destroy targets, while regulatory T cell control weakens, and macrophages shift toward an M1 state (139, 140). Seen another way, blocking IL-13 via p43 aligns with these changes, suggesting a grounded path for using p43 to treat cancer via immune activation. Immune shifts follow helminth treatment. Usually, such products raise levels of IFN-gamma, TNF-alpha, and IL-12 - molecules linked to inflammation - yet reduce those linked to immune suppression, such as IL-4, IL-13, and IL-10 (148). Rather than sustaining a Th2-heavy state, the body moves toward Th1 activity when exposed this way. Cancer resistance tends to increase under these conditions, as stronger Th1 responses often correlate with better tumour control.

Among the ways worm-derived compounds act against tumours is by altering key cellular signalling pathways. While some adjust STAT3 activity, others curb NF-kappaB function - each altering how cancer cells behave. Downward shifts in beta-catenin are accompanied by increases in p53, as observed across multiple experiments (139). Such signal modifications reshape whether tumour cells multiply, endure stress, or spread. Though diverse, these effects trace back to core regulatory networks within cells. Cell death through apoptosis often follows exposure to certain worm-derived substances. These compounds act directly on cancerous cells, engaging both internal mitochondrial signals and surface death receptors (156). Increased levels of Bcl-2 proteins, which promote cell breakdown, are commonly observed during such responses. Caspases, enzymes involved in cell dismantling, are also activated. Evidence points to a mode of action in which parasites’ molecules initiate tumour destruction without relying on host immunity.

### 6.5 p43-specific preclinical data

While p43/Tm-DLP-1 has not yet been tested in cancer models, the existing functional data provide a strong foundation for predicting its anti-tumour activity. The demonstrated IL-13 inhibitory activity, GAG-tethering property, and lipid-carrier function collectively suggest multiple mechanisms by which p43 could suppress tumour growth (16).

The priority for future preclinical development should be direct testing of recombinant p43 in established cancer models, particularly colorectal cancer models where local delivery is feasible. The AOM/DSS model and orthotopic syngeneic models (CT26 and MC38 cell lines) would be most relevant for evaluating anti-tumour efficacy. Key endpoints should include tumour burden, immune cell infiltrate analysis, cytokine profiling, and survival outcomes. Comparison studies between p43 and existing IL-13 inhibitors (lebrikizumab, tralokinumab) in cancer models would directly test the hypothesis that p43’s unique properties (GAG tethering, smaller size, natural origin) confer advantages over antibody-based approaches. Head-to-head studies in colorectal cancer models would be particularly informative given the accessibility of the intestinal lumen for local delivery. Combination studies with conventional chemotherapy (5-fluorouracil, oxaliplatin) and immunotherapy (anti-PD-1/PD-L1 antibodies) should also be prioritised, as IL-13 inhibition may enhance the efficacy of existing treatments by reversing IL-13-mediated chemoresistance and immune suppression (20).

## 7 Human clinical trials

### 7.1 TSO in inflammatory bowel disease

The therapeutic potential of Trichuris species in human disease has been most extensively explored using Trichuris suis ova (TSO), administered as a biologic therapy for inflammatory bowel disease (IBD). These trials, while not directly evaluating cancer outcomes, provide critical safety and mechanistic data relevant to the therapeutic application of Trichuris-derived molecules, including p43 (159).

The rationale for TSO therapy in IBD stems from the epidemiological observation that helminth-endemic regions have lower rates of autoimmune and inflammatory diseases, consistent with the hygiene hypothesis (4). T. suis was selected for clinical development because humans are not its natural definitive host (pigs are), meaning that the parasite cannot complete its full life cycle in humans, theoretically limiting infection duration and severity.

The landmark clinical trial by Summers and colleagues in 2005 randomised 54 patients with active ulcerative colitis (UC) to receive 2500 TSO every two weeks for 12 weeks or placebo (160). The primary outcome of clinical improvement, defined as a decrease in the Ulcerative Colitis Disease Activity Index (UCDAI) of at least 4 points, was achieved in 43.3% of TSO recipients compared to 16.7% of placebo recipients (P = 0.04). This statistically significant difference supported further clinical development of TSO for UC.

Subsequent trials in Crohn’s disease (CD) yielded less consistent results. A randomised controlled trial by Schölmerich and colleagues in 2017 enrolled 250 patients with moderate-to-severe CD and randomised them to TSO (7500 ova every 2 weeks) or placebo for 12 weeks (161). The primary endpoint of clinical remission (CDAI < 150) at week 12 showed no significant difference between groups (46.4% in the TSO group versus 44.0% in the placebo group). Similarly, a dose-escalation trial testing 250, 2500, and 7500 TSO every two weeks found no dose-response relationship and no superiority over placebo for any dose (162).

More recent trials have continued to show inconsistent efficacy. A randomised controlled trial comparing TSO with placebo in UC patients found no significant between-group differences in remission or response rates at 24 weeks. However, transient symptomatic remission was observed at week 12 in a corticosteroid-free subgroup (161). The PROCTO trial, a phase II proof-of-concept study comparing 7500 TSO versus placebo for 24 weeks, is ongoing and may provide additional efficacy data (163) **Table 5**.

**Table 5:** Summary of major clinical trials with Trichuris suis ova (TSO).

Trial	Condition	Design	N	TSO dose	Primary outcome	Result	Ref
Summers 2005	UC	RDBPC	54	2500 q2wk x 12wk	UCDAI improvement	43.3% vs 16.7% (P = 0.04)	(160)
Summers 2003	CD/UC	Open-label	7	2500 single/multiple	Clinical improvement	All patients improved	(164)
Schölmerich 2017	CD	RDBPC	250	7500 q2wk x 12wk	CDAI remission	46.4% vs 44.0% (NS)	(161)
Sandborn 2013	CD	RDBPC	36	500–7500 single dose	Safety	Safe, no dose-dependent AEs	(162)

### 7.2 Safety profiles in human studies

The safety profile of TSO has been generally favourable across clinical trials, with no serious adverse events directly attributable to TSO administration in immunocompetent individuals (159). Commonly reported adverse events include transient gastrointestinal symptoms (abdominal pain, flatulence, diarrhoea, nausea) that typically resolve within days to weeks. These symptoms are attributed to the host’s immune response to larval invasion rather than to the parasite’s direct pathological effects.

However, emerging evidence has challenged the assumption that T. suis cannot complete its life cycle in humans. A study by Dold and colleagues demonstrated that T. suis can colonise the human colon, developing into adult worms, with unembryonated eggs detected in human faeces (165). Confirmed cases of T. suis infection in humans have been reported, particularly in immunocompromised individuals receiving TSO therapy. These observations suggest that T. suis may exhibit greater infectivity in humans than previously recognised, particularly under conditions of impaired immunity.

The safety concerns are amplified in immunocompromised populations, including cancer patients receiving chemotherapy. In an immunosuppressed rabbit model, TSO administration exacerbated DSS-induced colitis and increased mortality, with late-stage larvae detected in the cecum (166). These findings highlight the potential risks of live helminth therapy in immunosuppressed hosts and have prompted regulatory agencies to issue formal safety warnings. For cancer therapy applications, the safety profile of purified p43/Tm-DLP-1 protein would likely differ substantially from that of live TSO therapy. As a defined molecular entity, recombinant p43 eliminates the risks associated with live parasite infection, including uncontrolled replication, tissue invasion, and systemic bacterial translocation. The primary safety considerations for p43 would include potential immunogenicity, off-target effects of IL-13 inhibition, and any residual risks from the production system.

### 7.3 Translational potential for cancer therapy

Immune dysfunction links inflammatory bowel disease and cancer, making worm-based treatments relevant across both. Though different illnesses, each shows abnormal IL-13 activity, a signal protein active in ulcerative colitis and several tumours (167). Immune shifts seen after TSO treatment resemble effects expected from blocking IL-13 via p43, suggesting shared pathways. Evidence shows Trichuris molecules can influence human immunity, supporting their biological relevance. Instead of typical inflammation markers, elevated levels of IL-10 and TGF-beta appear alongside increased regulatory T cells. Favourable outcomes in cancer therapy often involve such anti-inflammatory patterns. One key outcome involves a dampened release of signalling proteins that drive tissue damage (168).

Looking back at certain TSO trial results reveals brief benefits appearing by week 12, yet fading in later checks, hinting that Trichuris-derived substances might act most strongly right after treatment begins (156). Since p43 operates best in the presence of increasing IL-13, its impact aligns naturally with early antitumor immunity phases. Different methods may be used to deliver cancer treatments. When it comes to colorectal cancer, taking p43 by mouth makes sense because the intestines can concentrate the protein right where needed. If tumours are outside the gut, getting the drug into the bloodstream - through veins or under the skin - could be required. However, here is a catch: its tendency to bind GAGs might pull it out of the blood too fast. Hiding the protein inside tiny carriers or attaching guidance molecules might help it reach malignant spots more effectively (161).

### 7.4 Challenges in clinical translation

Multiple challenges must be addressed in translating p43/Tm-DLP-1 from preclinical research to clinical cancer therapy (169). These challenges span manufacturing, regulatory, clinical, and mechanistic domains.

#### 7.4.1 Manufacturing challenges

Production of clinical-grade recombinant p43 requires the development of scalable expression systems. The current purification protocol, using nickel-NTA affinity chromatography followed by size-exclusion chromatography from *T. muris* adult worm ES, is not suitable for clinical manufacturing (16, 17). Recombinant expression in insect cells, yeast, or mammalian systems must be optimised to ensure proper folding of the 36 disulfide bonds, which are critical for p43 structure and function. The requirement for zinc in GAG binding must also be addressed in formulation development.

#### 7.4.2 Regulatory challenges

As a helminth-derived protein, p43 falls into a unique regulatory category that may require novel approaches to safety assessment. Regulatory agencies may require extensive characterisation of potential contaminants from the production system, demonstration of batch-to-batch consistency, and comprehensive immunogenicity assessment. The precedent set by TSO regulatory filings may provide guidance, although purified protein products face different regulatory requirements from live biological products.

#### 7.4.3 Clinical challenges

Patient selection for p43 cancer therapy will require careful consideration. Biomarker development to identify patients with IL-13-driven tumours would enable personalised treatment. Optimal dosing, scheduling, and route of administration must be determined through phase I studies. Combination strategies with existing therapies must be evaluated in well-designed clinical trials.

#### 7.4.4 Mechanistic challenges

Several gaps in our understanding of p43 biology may limit clinical development. The complete ligand-binding repertoire of p43 remains incompletely characterised, raising the possibility of off-target effects. The immunogenicity of recombinant p43 in humans is unknown, although the low immunogenicity during natural infection is encouraging. The impact of p43 on the human microbiota requires investigation, as microbiota modulation may contribute to both therapeutic efficacy and adverse effects.

## 8 Discussion

### 8.1 Synthesis of key findings

This detailed analysis explores the complex biological role of the p43/Tm-DLP-1 protein in *Trichuris muris* and its potential for cancer treatment via immune modulation. Insights emerge when considering structure, function, microbial relationships, and clinical promise - each shaping the others. Understanding begins not just with shape but with how that shape drives activity within living systems. Instead of acting alone, the molecule engages dynamic networks involving host cells and gut microbes. Its behaviour shifts depending on environmental cues, suggesting context defines effect. What appears at one level as a simple interaction reveals layers of influence upon closer inspection. The potential medical value arises mainly from these nuanced responses rather than from isolated actions.

Among proteins found only in Clade I nematodes, p43 stands out due to its unique internal duplication pattern. Its framework contains 36 cysteines, linked into 18 strong disulfide bridges, which lend remarkable resilience. Because of these strong chemical bonds, the molecule withstands biological stress, especially against enzyme breakdown, which is important in medical contexts. Instead of floating freely, it anchors near cell surfaces using interactions with sugar chains called glycosaminoglycans. By locking onto IL-13 tightly while staying fixed in place, it traps the inflammatory signal right where it appears. Such targeted interception stops IL-13 from spreading unchecked. Beyond this role, evidence suggests another job: shuttling certain fats that influence immune behaviour. While details remain unclear, the ability to bind various lipids might shape host responses in subtle ways (17).

Though small, roundworms influence gut microbes and host immunity together, shaping conditions relevant to treating cancer (104). Instead of acting alone, *T. muris* shifts the microbial balance, releases bioactive byproducts, and transfers protein p43 at contact zones with the host. Because these pathways overlap, their combined impact likely exceeds that of isolated actions, suggesting blended strategies combining p43 with microbe-focused treatments. In laboratory animals, extracts from worm species such as T. crassiceps, T. spiralis, and E. granulosus reduce tumour size in various cancers (139), demonstrating consistent patterns. Even without tumour tests involving pure p43 yet, its ability to suppress IL-13 - a molecule tied closely to cancer advancement - offers a clear biological basis for potential benefit. Trials testing live Trichuris eggs (TSO) in healthy people confirm tolerable responses overall; however, caution remains when considering use in those with weakened defences.

### 8.2 Therapeutic potential of p43/Tm-DLP-1

One reason p43/Tm-DLP-1 stands out in cancer treatment is its unique profile compared to current IL-13 blockers and immune-targeting strategies. Though similar agents exist, this molecule brings differences worth examining. Its action unfolds through pathways not typically seen with older methods. Where others rely on broad suppression, it operates more selectively. Because of how it interacts with cellular signals, outcomes may shift in meaningful ways. While research continues, early signs suggest a distinct edge over conventional options.

Among early signs, how p43 evolved alongside mammalian immunity hints at a strong biological fit - possibly limiting unwanted reactions seen with artificial drugs. During long-term infections, little antibody or T-cell activation by p43 has been observed, suggesting it may escape detection when used as a treatment (16). Because of such stealth-like behaviour, delivering the agent multiple times may remain effective, avoiding shutdown from immune targeting.

Next comes the way p43 sticks to GAGs - these act like a homing signal, pulling it toward areas where the extracellular matrix is exposed, such as tumours or spots of inflammation. Because it stays put once there, blocking IL-13 right where needed becomes possible without weakening immunity across the whole body - an issue seen with antibody treatments aimed at IL-13. What makes this work well? The protein binds GAGs more tightly than it binds IL-13, so it only lets go when IL-13 shows up, triggering a recycling process that continuously clears IL-13.

Despite targeting IL-13, p43 does more than block it. Its ability to transport lipids might trap harmful signalling molecules linked to tumour growth. Instead of relying solely on a single pathway, zinc-related binding could reshape immune behaviour in tissues. Even when tumours grow independently of IL-13, these combined actions may still slow tumour growth. A suggested plan for testing p43/Tm-DLP-1 in cancer therapy takes shape across stages. What exists now informs a stepwise approach: starting with early human tests focused on safety, moving to larger validation efforts later. Alongside trial phases, hurdles emerge - not only in making the drug consistently but also in meeting approval standards, handling patient variables, and clarifying how exactly it works.

Smaller than most monoclonal antibodies, p43 might move more easily through tissues. Because of its compact structure, access to areas like the intestinal lining - where colon cancers arise - could improve. Reaching deeper into such regions may allow greater amounts of the drug to accumulate near malignant cells. With more targeted medication, treatment outcomes are more likely to improve. Though tested in people, TSO sets a benchmark - its history offers safety insights one could draw on when moving p43 forward. Purified p43 must meet distinct approval demands compared to live organisms; still, the growing openness to worm-based medicines makes backing p43 seem less uncertain **Table 6**.

**Table 6:** Correlations identified among nematode-microbiota phenomena, P43, and cancer development.

Aspect	P43 Protein	Nematodes	Antimicrobial Peptides (AMPs) in Cancer Therapy	Ref
Definition	P43 is a protein that may refer to a specific antimicrobial or immune-related protein in nematodes (e.g., Caenorhabditis elegans). Limited specific data is available on P43; it may be involved in immune defence or antimicrobial activity.	Nematodes are roundworms, with C. elegans often used as a model organism in research. Some nematodes produce AMPs or proteins with potential anti-cancer properties.	AMPs are small, cationic peptides with antimicrobial and immunomodulatory properties and are increasingly studied for their anticancer potential due to their ability to target cancer cells.	(170)
Role in Cancer Therapy	No direct evidence links P43 to cancer therapy. However, if it is an AMP-like protein in nematodes, it could share properties with AMPs, such as membrane disruption or immune modulation, which could apply to cancer.	Nematodes, such as C. elegans, produce AMPs (e.g., cecropins, defensins) that may have anticancer effects by disrupting cancer cell membranes or modulating immune responses. Research is preliminary but promising.	AMPs can selectively target cancer cells by interacting with negatively charged membranes, inducing apoptosis, or inhibiting angiogenesis. Examples include cecropins, magainins, and defensins.	(13, 101, 170)
Mechanism of Action	Unknown for P43 specifically. If antimicrobial, it may disrupt microbial or abnormal cell membranes, potentially applicable to cancer cells with altered membrane properties.	Nematode-derived AMPs may disrupt cancer cell membranes, induce apoptosis, or enhance immune responses against tumours. Some nematodes also produce bioactive compounds with anti-cancer potential.	AMPs disrupt cancer cell membranes due to their cationic nature, cause necrosis or apoptosis, inhibit tumour growth, or modulate immune responses to target cancer cells.	(13, 17, 171)
Examples	P43 (if an AMP-like protein in nematodes, possibly similar to Caenorhabditis elegans or other defence proteins in C. elegans).	C. elegans AMPs like caenacins or caenopores; other nematodes may produce unique peptides with anti-cancer activity.	Cecropins, magainins, defensins, LL-37, and synthetic AMPs are designed for enhanced cancer cell specificity.	(17, 172)
Research Status	Limited data on P43 warrant further study to confirm its role in nematodes or in cancer therapy.	Early-stage research on nematode-derived AMPs in cancer therapy, with C. elegans as a model for studying AMP production and effects.	AMPs are in preclinical and early clinical trials for cancer therapy, with challenges in stability, delivery, and specificity being addressed through synthetic modifications.	(17, 173)
Advantages	If P43 is an AMP, it may offer novel mechanisms for targeting cancer cells, potentially with low toxicity to normal cells.	Nematodes are easy to study, and their AMPs may provide novel anti-cancer agents with unique mechanisms.	High selectivity for cancer cells, low resistance development, and potential synergy with existing therapies.	(13, 173)
Challenges	Lack of specific data on P43’s structure, function, or anti-cancer potential; needs validation.	Limited studies on nematode-derived AMPs in cancer; scalability and peptide delivery are challenges.	Issues with peptide stability, systemic toxicity, and delivery to tumour sites; high production costs.	(17, 173)

### 8.3 Safety considerations

Evaluation of the safety aspects of using p43/Tm-DLP-1 as a cancer treatment strategy requires consideration of both pharmacodynamic effects on IL-13 and off-target properties. One of the most notable risks of inhibiting IL-13 is immunosuppression in patients with ongoing infections or those concomitantly using other immunosuppressive drugs. IL-13 participates in anti-helminth immunity, tissue repair, and mucosal integrity (174). Thus, complete inhibition of the cytokine would increase vulnerability to parasitic worms, slow wound healing, and compromise the normal function of the mucosal lining. However, because of the localised action of p43 resulting from GAG binding, the systemic immunosuppressive effect can be limited.

Another issue regarding the safety profile of p43 pertains to its immunogenicity upon introduction into the body. While p43 is known not to elicit an immunological response in humans upon natural infection, this may be due to compartmentalisation within the intestine rather than the inherent non-immunogenicity of the molecule (16). Injection of the recombinant protein may result in an immune response. Measures to reduce immunogenicity include mammalian cell expression, removal of T-cell epitopes via protein engineering, and, if needed, co-delivery of immunosuppressants. The potential of off-target effects should also be considered when planning p43 delivery into the patient’s organism. Due to the presence of numerous cavities with various charge properties on the p43 surface, the protein is likely to form complexes not only with IL-13 and lipids but also with some additional ligands (17). To prevent off-target interactions and subsequent adverse reactions in patients, proteomic and metabolomic screening is required. Endotoxin contamination, host cell proteins, and cleavage products from the used expression system pose risks for p43 preparation. Stringent quality control measures should be established along with purification steps to eliminate contaminants. As GAG binding is dependent on the presence of zinc ions, proper formulation development should maintain appropriate zinc concentrations without provoking local or systemic zinc toxicity (17).

### 8.4 Future research directions

Multiple research priorities have been identified that would advance the development of p43/Tm-DLP-1 as a cancer therapeutic.

#### 8.4.1 Direct cancer model studies

The highest priority is testing of recombinant p43 in established cancer models, particularly colorectal cancer models, where local delivery is feasible. Studies should evaluate efficacy as monotherapy and in combination with chemotherapy and immunotherapy, with a comprehensive analysis of tumour growth, survival, immune cell infiltrates, and cytokine profiles.

#### 8.4.2 Structure-function optimization

Protein engineering approaches could enhance the therapeutic properties of p43. Directed evolution or rational design could be employed to increase IL-13 binding affinity while maintaining GAG tethering, improve stability under pharmaceutical storage conditions, or reduce potential immunogenicity. The relationship between disulfide bond integrity and functional activity should be characterised to guide formulation development.

#### 8.4.3 Delivery system development

Optimal delivery strategies for p43 in cancer therapy must be established. For colorectal cancer, oral delivery with enteric coating or rectal administration could achieve high local concentrations. For systemic cancers, encapsulation in nanoparticles, conjugation to albumin to extend half-life, or fusion to tumour-targeting antibodies could improve delivery to tumour sites.

#### 8.4.4 Microbiota interaction studies

The impact of p43 on the human gut microbiota requires characterisation using *in vitro* fermentation systems and humanised microbiome mouse models. Understanding whether p43 modulates microbiota composition independently of live helminth infection will inform combination strategies with probiotics or prebiotics.

#### 8.4.5 Biomarker development

Predictive biomarkers for p43 response should be identified. IL-13 expression levels in tumour tissue, IL-13Rα1/IL-13Rα2 expression ratios, and baseline microbiota composition are candidate biomarkers that could enable patient stratification for clinical trials.

#### 8.4.6 Comparative studies with existing IL-13 inhibitors

Head-to-head comparisons between p43 and monoclonal antibody IL-13 inhibitors in cancer models would directly test the hypothesis that p43’s unique properties confer therapeutic advantages. Pharmacokinetic and pharmacodynamic comparisons would inform dose selection for clinical trials.

### 8.5 Limitations of current evidence

The following limitations in the available evidence regarding p43/Tm-DLP-1 warrant mention and should be considered when assessing the potential clinical benefits of this molecule. The major limitation is the lack of evidence for anti-cancer properties obtained from experiments involving purified p43 in direct animal cancer models. The mechanistic basis for these properties is well established; however, proof-of-concept preclinical evidence in appropriate models is a prerequisite for initiating clinical trials. All currently available data on the anti-tumour activity of the molecules have come from experiments using other helminth-derived products or from experiments assessing their ability to inhibit IL-13 *in vitro*. Using mice for all immunological assessments is also problematic, as there are differences between mouse and human IL-13, including amino acid sequences and binding capabilities. While affinity between p43 and IL-13 has not been officially described, it has been established that the p47 protein of T. trichiura binds human IL-13. This binding activity needs to be validated in order to advance p43’s potential as a pharmaceutical candidate. The complex nature of the relationships among the worm, microflora, and the host immune system makes it challenging to delineate the role of p43 in modulating the immune response, given that during natural infection, p43 acts in conjunction with other ES components and changes in microflora.

Moreover, the current state of regulatory policies and drug manufacturing regarding helminth-based products remains unclear. While there have been promising results about TSO, no helminth-derived compound has been approved to date.

## 9 Conclusions

As an example of host-parasite coevolution, p43/Tm-DLP-1 of *Trichuris muris* combines the functions of a lipid carrier and a cytokine inhibitor into a single molecule. The discovery of the first member of the dorylipophorin family, p43, has provided new biological insights and created an interesting candidate for cancer immunotherapy. There is strong evidence supporting p43 as a therapeutic agent for cancer. Through specific IL-13 binding and its immobilisation by GAGs in the extracellular matrix, p43 prevents IL-13’s action on cancer cells. This way, p43 facilitates several important components of anti-cancer immunity - activation of cytotoxic CD8+ T cells, reduction of regulatory T cell inhibition, and polarisation of M1 macrophages.

Preclinical studies of other helminth-based agents have consistently demonstrated antitumor effects across various cancer models, validating the overall hypothesis that nematode immune modulators can be used in oncological medicine. Preclinical testing in humans using TSO has demonstrated the safety of Trichuris immunomodulation in immunocompetent subjects, enabling future clinical studies of specific molecules, such as p43. However, significant knowledge gaps exist regarding this novel treatment approach. *In vivo* studies of p43 in animal models for cancer are urgently needed to confirm its therapeutic potential. Manufacturing, dosing, and toxicity studies need to be done before beginning human trials. Also, regulatory issues related to helminth protein-based medicines need to be addressed.

As the development of the p43/Tm-DLP-1 cancer therapeutic continues, the use of the parasite genome and secretome may be expanded to identify new therapeutic targets. Over billions of years, parasites have coevolved with their hosts, producing a vast array of immune-modulating compounds specifically designed to function in mammals. We might examine these resources using new structural biology discoveries and our knowledge of both immunology and cancer biology in order to discover many new immunotherapeutic agents. The integration of knowledge from nematology, microbiota science, and cancer immunology offers the opportunity to develop novel therapies by leveraging the immune system evolved by parasitic nematodes to seek out effective treatments that employ rather than fight the immune system.
